# Evaluating the Discriminative Performance of Noninvasive Biomarkers in Chronic Hepatitis B/C, Alcoholic Cirrhosis, and Nonalcoholic Cirrhosis: A Comparative Analysis

**DOI:** 10.3390/diagnostics15131575

**Published:** 2025-06-20

**Authors:** Alina Dumitrache (Păunescu), Nicoleta Anca Ionescu (Șuțan), Monica Marilena Țânțu, Maria Cristina Ponepal, Liliana Cristina Soare, Ana Cătălina Țânțu, Muhammed Atamanalp, Ileana Monica Baniță, Cătălina Gabriela Pisoschi

**Affiliations:** 1 University of Medicine and Pharmacy of Craiova, Petru-Rareș Street No. 2, 200349 Craiova, Romania; alina.paunescu@upb.ro (A.D.); catalina.tantu8@gmail.com (A.C.Ț.); monica.banita@yahoo.com (I.M.B.); c_pisoschi@yahoo.com (C.G.P.); 2 Department of Natural Sciences, National University of Science and Technology Politehnica Bucharest, Piteşti University Centre, 1st Targu din Vale Str., 110040 Pitesti, Romania; maria.ponepal@upb.ro (M.C.P.); liliana.soare@upb.ro (L.C.S.); 3 Department of Medical Assistance and Physical Therapy, National University of Science and Technology Politehnica Bucharest, Piteşti University Centre, 1st Targu din Vale Str., 110040 Pitesti, Romania; 4 Department of Aquaculture, Faculty of Fisheries, Ataturk University, TR-25240 Erzurum, Turkey; mataman@atauni.edu.tr

**Keywords:** chronic hepatitis B, chronic hepatitis C, alcoholic cirrhosis, nonalcoholic cirrhosis, direct biomarkers, indirect biomarkers

## Abstract

**Introduction:** The clinical implementation of noninvasive tests for liver fibrosis assessment has attracted increasing attention, particularly for diagnosing advanced fibrosis (≥F3). This observational study aimed to evaluate the stratification accuracy of nine direct and seven indirect biomarkers across four etiologies: chronic hepatitis B (CHB), chronic hepatitis C (CHC), alcoholic liver cirrhosis (ALC), and nonalcoholic liver cirrhosis (NALC). **Materials and Methods**: Our study was conducted on 116 participants, including 96 with chronic liver disease (16 CHB, 15 CHC, 49 ALC, and 16 NALC) and 20 healthy controls. The values of direct (aspartate aminotransferase, alanine aminotransferase, total bilirubin, serum albumin, platelet count, international normalized ratio, gamma-glutamyl transpeptidase, CD5 antigen-like, and transforming growth factor-beta 1) and indirect non-serological biomarkers (De Ritis ratio, albumin–bilirubin score, gamma-glutamyl transpeptidase-to-platelet ratio, aspartate aminotransferase-to-platelet-ratio index, fibrosis-4 index, INR-to-platelet ratio, and fibrosis quotient) were analyzed for their discriminative power in fibrosis stratification. **Results:** Statistical analyses revealed a significant correlation (0.05 level; two-tailed), and AUC 95% CI ranged within 0.50–1.00 between the direct and indirect biomarker values across all etiologies. Among the evaluated biomarkers, the recorded AUC was 0.998 in CHB for APRI, 0.981 in CHC for FIB-4, and 1.000 in ALC and NALC for APRI and AST, respectively, while CD5L consistently achieved an AUC of 1.000 across all etiologies. **Conclusions:** These findings suggest that applying a multifactorial approach in liver pathology may improve diagnosis accuracy compared to the use of individual biomarkers and can provide data that may inform the development of clinically applicable mathematical models.

## 1. Introduction

Liver fibrosis is a progressive and dynamic process that is considered precancerous, and it is characterized by the formation of scar tissue as a response to chronic liver damage secondary to various etiologies (viral infections with virus B or C, alcohol abuse, or liver steatosis). Fibrotic lesions cause the growth of the extracellular matrix, with evolution towards liver cirrhosis, an irreversible condition that can result in liver failure and serious complications [1].

**Figure 1:**
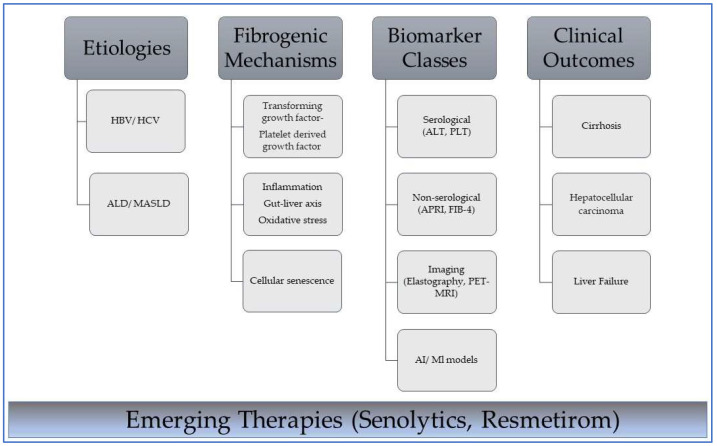
Interplay between etiology, mechanisms, diagnostic tools, and outcomes in liver fibrosis. HBV—hepatitis B virus; HCV—hepatitis C virus; ALD—alcohol-associated liver disease; MASLD—metabolic dysfunction-associated steatotic liver disease. Dark gray boxes indicate primary classification headers in the disease progression framework from cause to clinical manifestation; light gray boxes indicate subcategory items under each primary category and specific examples of biomarker classes. Lines connect subcategories to categories. The blue gradient box highlights the current focus on emerging therapeutic approaches that intersect with the biological and clinical components of the pathway. These therapies represent future interventions that can disrupt or modify these pathways at multiple stages.

Persistent injury triggers a cascade of fibrogenic mechanisms, such as TGF-β1-mediated extracellular matrix deposition, oxidative stress-induced apoptosis, and chronic inflammation driven by cytokines such as IL-6 (interleukin 6) and TNF-α (tumor necrosis alfa), which collectively promote excessive extracellular matrix deposition. Recent studies highlight the role of cellular senescence and senescence-associated secretory phenotypes (SASPs) in promoting fibrogenesis through paracrine signaling [2], as well as the role of gut microbiota dysbiosis in exacerbating inflammation via the gut–liver axis [3]. These processes culminate in cirrhosis, a terminal stage marked by architectural distortion, portal hypertension, and a heightened risk of hepatocellular carcinoma [4]. The dynamic interplay between disease drivers and diagnostic–clinical trajectories is illustrated in Figure 1.

Globally, chronic liver disease and cirrhosis remain a leading cause of mortality, accounting for over 2 million annual deaths [5,6]. In Europe, cirrhosis prevalence reflects significant regional disparities. Predominant etiologies include HBV/HCV [7], alcohol-associated liver disease, and nonalcoholic fatty liver disease, with the latter now affecting 25% of adults globally due to rising obesity and metabolic syndrome [8]. The most well-known etiologies in liver cirrhosis are HBV, HCV, alcohol-related liver disease (ALD), and nonalcoholic fatty liver disease [9].

Early detection of the degree of fibrosis (moderate or advanced) has significant clinical benefits, as it enables the identification of patients in early stages before symptoms manifest, allowing timely intervention to slow or prevent disease progression [10]. Noninvasive methods, such as transient elastography (FibroScan^®^, ECHOSENS, Paris, France) and biomarker panels (FIB-4 and APRI), remain foundational in early fibrosis detection [11,12]. Recent studies have shown that, in liver pathology, CD5L levels are elevated in patients with hepatic injury [13] and are associated with hepatic fibrosis [1] and cancer [14]. Moreover, they are upregulated during acetaminophen hepatotoxicity [15]. Notably, CD5L deficiency attenuates liver damage by reducing the activation of c-Jun N-terminal kinase and extracellular signal-regulated kinase signaling pathways, suggesting a pathogenic role in oxidative stress-mediated hepatocellular injury and immune activation [15,16]. However, advancements in artificial intelligence (AI)-enhanced models, such as machine learning (ML) algorithms that integrate multi-omics data, now enable higher precision in fibrosis stratification [17]. Emerging imaging modalities and targeted antifibrotic therapies further expand the diagnostic and therapeutic landscape [18,19].

Direct biomarkers (class I) are associated with the processes of synthesis and the degradation of the extracellular matrix, while indirect biomarkers (class II) reflect the degree of liver function and are used as predictive biomarkers of fibrosis and cirrhosis [20,21].

This study systematically compares serological biomarkers (ALT, AST, ALB, TBIL, PLT, PDW, MPV, INR, CD5L, TGF β1, and GGT) as direct indicators and non-serological biomarkers (AST/ALT ratio, ALBI score, GPR, APRI, INPR, FIB-4, BMI, and FibroQ) as indirect indicators of liver fibrosis across four distinct etiologies—CHB, CHC, ALC, and NALC—highlighting etiology-specific patterns and addressing heterogeneity in fibrosis mechanisms. We aim to evaluate their diagnostic accuracy given the etiological heterogeneity and varying fibrosis mechanisms, with the goal of establishing a reliable, noninvasive biomarker panel that can reduce the need for liver biopsy. We raise the following question: Can a combined panel of direct and indirect serological biomarkers accurately differentiate significant fibrosis and cirrhosis stages in patients with diverse chronic liver diseases? The primary endpoint is the diagnostic accuracy—measured via the area under the receiver operating characteristic curve (AUC)—of these biomarkers in distinguishing significant fibrosis and cirrhosis.

## 2. Materials and Methods

### 2.1. Study Design and Ethical Considerations

The hospital-based observational study protocol was approved by the Scientific Ethics and Deontology Commission of the University of Medicine and Pharmacy of Craiova (nr.48/29 January 2024), and all procedures were conducted with strict adherence to the ethical principles of the Declaration of Helsinki. Informed consent was obtained from all participants prior to inclusion in this study. Individuals who refused to provide consent were not included in this study.

#### 2.1.1. Inclusion and Exclusion Criteria

The eligible participants included adults diagnosed with chronic hepatitis (B/C), ALC, or NALC. The exclusion criteria comprised comorbid cardiovascular, cerebrovascular, and renal diseases or uncontrolled endocrine disorders.

#### 2.1.2. Participant Selection and Grouping

This study enrolled 116 participants, including 96 patients with chronic liver diseases (30 women and 66 men) and 20 healthy controls (10 women and 10 men). The participants were recruited from the Gastroenterology Service of the Pitesti County Hospital, Argeș County, Romania, where they presented themselves between January and June 2024.

For each participant included in this study, the following demographic and clinical data were collected: age, gender, height, weight, body mass index (BMI), and etiology of chronic liver disease. While not a biomarker per se, age represents an important non-modifiable risk factor for fibrosis progression. The body mass index (BMI) was calculated according to the following formula:

$$BMI=\frac{weight\;\left(kg\right)}{{height}^{2}\;\left(m^{2}\right)}$$

The study groups were formed following specific selection criteria.

The selection criteria for the control group (*n* = 20) were as follows: (I) no present liver diseases in their history (viral hepatitis, cirrhosis, or neoplasm); (II) normal abdominal ultrasound findings.

Sixteen patients exhibited chronic hepatitis B (CHB), and their diagnosis required the following: (I) liver dysfunction, such as elevated liver enzymes (AST/ALT ≥ 1.5× upper limit of normal [ULN]); (II) positive hepatitis B surface antigen (HBsAg).

Fifteen patients exhibited chronic hepatitis C (CHC). Their diagnosis was confirmed by the following: (I) liver dysfunction, such as elevated liver enzymes (AST and ALT); (II) positive anti-hepatitis C virus (HCV) antibodies; (III) positive outcome upon HCV ribonucleic acid amplification testing.

Forty-nine patients exhibited alcoholic liver cirrhosis (ALC), which included the following: cirrhosis confirmed via (I) hepatic dysfunction; (II) liver cirrhosis detected using FibroScan; (III) a long history of alcohol intake with a consumption threshold of 20–30 g alcohol/day, consistent with the EASL Clinical Practical Guidelines [22].

Sixteen patients exhibited non-alcoholic liver cirrhosis (NALC). The diagnosis required the following: (I) hepatic dysfunction; (II) liver cirrhosis detected via FibroScan; (III) a long history of steatohepatitis.

### 2.2. Laboratory Investigations

#### 2.2.1. Direct Serological Tests

This study employed direct serological tests to quantify class I biomarkers associated with hepatic injury, which included the following: alanine aminotransferase (ALT), aspartate aminotransferase (AST), albumin (serum), gamma-glutamyl transpeptidase (GGT), total bilirubin, platelet count (PLT), international normalized ratio (INR), platelet distribution width (PDW), mean platelet volume (MPV), CD5 antigen-like (CD5L), and transforming growth factor-beta 1 (TGF-β1). The values of the parameters investigated were taken from the medical records of the patients included in this study, except for CD5L/TGF-β1, which was measured prospectively. Discriminatory values were present between groups rather than definite diagnoses.

#### 2.2.2. Sample Collection and Handling

For each participant, two blood samples were collected for hematologic and biochemical measurements. Blood samples were collected after 12 h of fasting in commercially available red cap test tubes without anticoagulants and in K_2_EDTA tubes to prevent coagulation. The samples collected in K_2_EDTA tubes were used to perform complete blood cell counting (CBC). To obtain serum, blood was allowed to clot at room temperature and then separated through centrifugation at 1000× *g* for 10 min and used for the determination of biochemical biomarkers.

#### 2.2.3. Hematological and Biochemical Investigations

Complete blood cell counting was performed in peripheral venous blood samples obtained via venipuncture in K_2_EDTA vacutainers using an automatic hematological analyzer (Mindray BC-6800, Nanshan District, Shenzhen, China). From the hematological data, we determined the platelet number (PLT), platelet distribution (PDW), and median platelet volume (MPV). The coagulation tube was also used to calculate the INR value. Biochemical markers (ALT, AST, GGT, albumin, and total bilirubin) were assessed using an automated analyzer (Thermo Scientific Konelab Prime 30i, BioTeknics Leboratory Equipment, Les Pennes-Mirabeau, France). The enzymatic activity of AST and ALT was determined using standard kits: MAK467 for AST and MAK052 for ALT (Sigma-Aldrich, St. Louis, MO, USA). All transaminase ratios were calculated as AST/ALT to maintain clinical convention. For the determination of GGT, the GGT Colorimetric Assay Kit MAK089 (Sigma-Aldrich, USA) was used, and for the determination of serum albumin, the serum albumin ELISA Kit RAB0603 (Sigma-Aldrich, USA) was used. Total bilirubin was determined using the Hematoidin Assay Kit MAK126 (Sigma-Aldrich, USA). All tests were performed according to the manual provided with the assay kit.

#### 2.2.4. Serological Determination

CD5L and TGF-β1 levels were determined in duplicate using the Enzyme-Linked Immunosorbent Assay (ELISA) immunological method. CD5L was determined using a specific sandwich ELISA kit (RAB1347-1KT, Sigma-Aldrich, USA) for serum, plasma, and cell supernatant, according to the manufacturer’s instructions.

The CD5L test had a sensitivity of 0,5 pg/mL. The intra-assay and inter-assay coefficients of variation were <10% and <12%, respectively. No significant cross-reactivity or interference between CD5L and analogs was observed. The absorbance measurements were performed at 450 nm using a StatFax 4200 dual-wavelength microplate reader.

TGF-β1 was determined using the TGF-β1 ELISA kit (EIA-1864, DRG Instruments GmbH, Marburg, Germany) according to the manufacturer’s instructions. The analytical sensitivity was found to be 3.35 pg mL^−1^. The intra-assay and inter-assay coefficients of variation were 3.9–8.0% and 4.0–6.7%, respectively. Hemoglobin (up to 4 mg mL^−1^), bilirubin (up to 0.5 mg mL^−1^), and triglycerides (up to 7.5 mg/mL) had no influence on the assay’s results. The tests were quantified at 450 nm using a dual-wavelength microplate reader: StatFax 4200 (Awareness Technology, Palm City, FL, USA).

#### 2.2.5. Indirect Serological Assessments

Seven indirect class II biomarkers associated with fibrogenesis and cirrhosis were employed as indirect serological tests: the AST-to-platelet ratio index (APRI), fibrosis-4 index (FIB-4), albumin–bilirubin (ALBI) score, gamma-glutamyl-transpeptidase-to-platelet ratio (GPR), AST/ALT ratio (De Ritis ratio), INR-to-platelet ratio (INPR), and fibrosis quotient (FibroQ). The results are reported as means ± standard deviations and relative to the minimum–maximum range. The following biomarker formulas were applied:

APRI [23]

$$APRI=\frac{\frac{AST\;\left(U/L\right)}{ULN\;for\;AST(48\;U/L)}}{PLT\;count\;(\times 10^{3}/\mu L)}\times 100$$

FIB-4 [12] $FIB-4=\frac{Age\;\left(yr\right)\times AST\;(U/L)}{PLT\;count\times \sqrt{ALT\;(U/L)}}$

GPR [24] $GPR=\frac{\frac{GGT\;(U/L)\times 100}{ULN\;for\;GGT\;(55\;U/L)}}{PLT\;count\;(\times 10^{3}/\mu L)}$

The ALBI score [25] was calculated to establish the degree of liver function:

$$ALBI\;SCORE=0.66\times \log_{10}bilirubin\;(L^{-1})-0.085\times albumin(gL^{-1})$$

The AST/ALT ratio (De Ritis ratio) was calculated as the ratio between the concentrations of the hepatocytic enzymes aspartate aminotransferase (AST) and alanine aminotransferase (ALT) in the human blood [26]. The values of this ratio were determined only in patients who exhibited elevated AST and ALT values.

The INPR (INR-to-platelet ratio) is an index introduced to amplify the difference between the INR and the number of platelets [27], with a potential predictive value relative to the degree of fibrosis:

$$INPR=\frac{INR}{PLT\;count(\times 10^{3}/\mu L)}\times 100$$

FibroQ (fibrosis quotient) is an index that substitutes positive correlation parameters—such as age, INR, and AST—in the numerator and negative correlation parameters—such as number of platelets and ALT—in the denominator [28]:

$$FibroQ=10\;\text{×}age(year)\times AST(UI/L)\times INRPLT\;count(\times 10^{3}/\mu L)\times ALT(UI/L)$$

All biomarker values were interpreted relative to both laboratory reference ranges and established clinical thresholds (Table 1).

### 2.3. Statistical Analysis

The direct and indirect serological biomarker values are expressed as follows: means ± standard deviations (SDs); minimum–maximum range to summarize central tendency; and variability. Normally distributed variables were compared using independent-sample t-tests; non-normally distributed variables were analyzed with Mann–Whitney U tests. Given the samples’ sizes (*n* = 15–49 per group), a post hoc power analysis was conducted using G*Power 3.1 based on the observed effect sizes (Cohen’s *d* = 0.595).

Bonferroni-adjusted post hoc tests were conducted to control the family-wise error rate across multiple pairwise comparisons. The significance threshold (α) was adjusted to *α* = 0.05/*k*, where *k* denotes the number of pairwise comparisons per dependent variable, thereby reducing Type I error inflation. The adjusted *p* < 0.05 values were deemed statistically significant, with mean differences reported with 95% confidence intervals (CIs).

ROC curve analyses were employed to evaluate the discriminative performance of biomarkers and predictive models, quantifying the trade-off between sensitivity (true positive rate) and specificity (1—false positive rate) across different classification thresholds. The area under the curve (AUC) served as the primary metric, with the values interpreted as follows: (1) AUC = 1.0: perfect discrimination; (2) AUC = 0.5: no discriminative capacity (equivalent to random chance).

A total of 96 variables were assessed for their ability to distinguish between positive and negative clinical outcomes. The key parameters, including AUC, sensitivity, and specificity, were computed for each variable. The results were stratified to identify variables with optimal (highest AUC) and suboptimal (lowest AUC) discriminative utility.

ROC curve analyses were performed with calculations of the 95% confidence intervals via bootstrap resampling. AUC comparisons were carried out using DeLong’s test for correlated ROC curves. All perfect AUC values (1.00) were explicitly examined for potential overfitting.

The Kruskal–Wallis test was applied to evaluate median differences across five independent clinical cohorts: control, CHB, CHC, ALC, and NALC. The following continuous variables were analyzed: liver function markers: ALT, AST, GGT, total bilirubin, and albumin; hematologic indices: PLT, PDW, MPV, and INR; fibrogenesis/cirrhosis biomarkers: CD5L and TGF-β1; composite scores: AST/ALT ratio, ALBI score, GPR, APRI, INPR, FIB-4, and FibroQ; anthropometric measures: body mass index (BMI). All analyses rejected the null hypothesis, indicating statistically significant heterogeneity in biomarker distributions between outcome groups.

**H_0_.**  “*the distribution of [biomarker] is the same across categories of OUTCOME*”

Boxplots were employed to visualize data distribution, characterized by a bold central line representing the median, an interquartile range (IQR) bounded by the 25th and 75th percentiles at the box’s lower and upper limits, and whiskers extending vertically to adjacent values (minimum and maximum within 1.5 × IQR). Mild outliers, defined as observations beyond 1.5 × IQR but within 3 × IQR, were denoted by “o”, while extreme outliers (>3 × IQR) were marked with “*”. A two-tailed alpha threshold of 0.05 (*p* < 0.05) was used to define statistical significance throughout the analyses.

Missing data were excluded listwise (complete-case analysis). No imputation or advanced methods were applied to address missing values. All analyses were performed using IBM SPSS Statistics for Windows (Version 20.0; IBM Corp., Armonk, NY, USA) [29].

## 3. Results

### 3.1. Comparison of the General Characteristics Between Groups

Our study group comprised 116 participants, including 96 patients diagnosed with chronic liver diseases and stratified according to etiology (CHB, *n* = 16; CHC, *n* = 15; ALC, *n* = 49; NALC, *n* = 16) and 20 age-matched and sex-matched healthy controls recruited to establish baseline parameters. Statistical analyses revealed significant deviations from the test value (*p* < 0.05). The large effect sizes (Cohen’s d > 1.5) indicate that the mean of this variable is approximately 1.6 standard deviations above the test value. The wide confidence intervals (5.13–41.27) reflect uncertainty in the true effect’s magnitude, and this phenomenon is likely due to the small sample size. Post hoc power analyses (α = 0.05, effect sizes = 1.5–1.9) suggested moderate power (60–70%) in detecting large effects, but insufficient power (<80%) for smaller effects. For the smallest group (*n* = 15), the power ranged from 65 to 75% when detecting large effects (d > 1.5). In contrast, for the largest group (*n* = 49), the power exceeded 95% for large effects but decreased to 40–50% for small-to-moderate effects (d = 0.3–0.5).

Comprehensive demographic and clinical characteristics, including age, sex, BMI, and etiology-specific risk factors, are summarized in Table 2.

CHB (69%), CHC (60%), and ALC (78%) were characterized by male predominance, whereas NALC exhibited equal gender distribution. For the CHB cohort, the mean recorded age was 51.3 ± 14.3 years, with these patients being younger than the ALC cohort, who had a mean age of 57.37 ± 10.08 years, although there were no significant differences. The BMI in the case of NALC patients registered statistically significantly higher values compared to the control group (*p* < 0.05); they fell within the first degree of obesity, with a BMI varying between 30 and 34.99. Patients in the control and CHB groups fell into the category of overweight, with a BMI value between 25 and 29.9. In comparison, patients in the CHC and ALC groups exhibited a slight decrease in BMI, which ranged from 18.5 to 24.99, falling within the normal weight category.

### 3.2. Comparison of Direct Serum Biomarker Values Between Groups

Direct serum biomarkers demonstrated significant variations across study groups (Table 3). AST and ALT levels increased progressively from the control to cirrhotic groups, with NALC exhibiting the highest values (AST = 111.75 ± 49.95 UI/L; ALT = 57.43 ± 34.21 UI/L). The Bonferroni-adjusted post hoc analysis (Table S1) confirmed significant differences in the biomarker profiles across the study groups (Control, CHB, CHC, ALC, and NALC), reflecting the distinct pathophysiological signatures of chronic hepatitis B/C, ALC, and NALC. The NALC and ALC groups exhibited markedly elevated ALT and AST levels compared to the control group (ALT: NALC Δ = −33.29, *p* < 0.001; ALC Δ = −22.23, *p* = 0.004; AST: NALC Δ = −95.55, *p* < 0.001; ALC Δ = −83.66, *p* < 0.001). No significant differences (*p* = 1.0) were observed for these biomarkers when CHB/CHC was compared to the control group.

Hypoalbuminemia and hyperbilirubinemia were pronounced in the cirrhotic group, reflecting impaired synthetic function. Albumin levels in NALC (23.96 ± 4.00 g dL^−1^, Δ = −18.39, *p* < 0.001) were markedly reduced compared to the control group (42.35 ± 5.11 g dL^−1^), while the total bilirubin surged to 332.01 ± 253.12 µmol L^−1^ in NALC versus 52.60 ± 15.83 µmol L^−1^ in the control group. Thrombocytopenia was the most severe in NALC (115.06 ± 70.70 × 10^9^ L^−1^).

Comparative analyses using the independent-sample *t*-test revealed distinct biomarker profiles across etiological groups. Based on the Bonferroni-adjusted post hoc analysis, NALC exhibited a statistically significant highest TBILI (Δ = −279.41 vs. Control, *p* < 0.001), followed by ALC (Δ = −147.22, *p* = 0.001), reflecting severe hepatic synthetic dysfunction in cirrhosis. In CHB patients, ALT, total bilirubin, and GGT levels demonstrated no statistically significant differences relative to the control group. Conversely, the chronic hepatitis C group (CHC) exhibited non-significant variations in ALT and INR values. NALC cases exhibited significant deviations in all biomarkers, with the exception of INR, which remained comparable to the control group’s values. Strikingly, ALC patients exhibited statistically significant differences (*p* < 0.05) across all direct biomarker measurements compared to the control group.

CD5L was depleted in all disease groups when compared to the control group (NALC Δ = −9.12, *p* < 0.001; ALC Δ = −9.11, *p* < 0.001), while TGF-β1 was elevated in NALC (Δ = +67.94, *p* < 0.001) and ALC (Δ = +71.85, *p* < 0.001).

The Bonferroni-adjusted post hoc analysis revealed prolonged coagulation in cirrhosis (ALC Δ = +0.36, *p* < 0.001; NALC Δ = +0.26, *p* = 0.019), and thrombocytopenia was pronounced in NALC (Δ = −182.34, *p* < 0.001) and ALC (Δ = −156.45, *p* < 0.001), reflecting portal hypertension and splenic sequestration.

GGT was significantly elevated in ALC (Δ = −145.69, *p* = 0.012) but not in NALC (*p* = 0.085), highlighting alcohol-specific toxicity.

### 3.3. Comparison of Non-Serological Biomarker Values Between Groups

The independent-sample T-test (*p* < 0.05) demonstrated that all non-serological biomarkers exhibited statistically significant differences relative to the control group. The AST/ALT ratio markedly escalated relative to disease severity, with the control group (0.67 ± 0.12) exhibiting the lowest values, followed by CHB = 1.14 ± 0.27 and CHC at 1.23 ± 0.29, which finally peaked in ALC = 2.06 ± 0.82 and NALC = 2.14 ± 0.77. The significantly higher AST/ALT ratio in cirrhosis (NALC Δ = +1.47, *p* < 0.001; ALC Δ = +1.39, *p* < 0.001) was consistent with the Bonferroni-adjusted post hoc analysis (Table 3). The ALBI score deteriorated progressively from the control to cirrhotic groups (NALC Δ = +2.04, *p* < 0.001; ALC Δ = +1.61, *p* < 0.001), while noninvasive fibrosis indices (APRI, FIB-4, INPR, and FibroQ) exhibited a pronounced increase from the health controls to those with advanced disease. Thus, APRI increased from 0.10 ± 0.02 in the control group to 2.75 ± 2.09 in NALC (APRI: NALC Δ = +2.65, *p* < 0.001), and FIB-4 increased from 0.62 ± 0.16 (control) to extreme values in NALC (10.63 ± 7.41, Δ = +10.01, *p* < 0.001). GPR and FibroQ reflected parallel trends, with NALC values (4.15 ± 5.22 and 17.31 ± 12.05, respectively) far exceeding the control group. Based on independent-sample T-tests for *p* < 0.05, all non-serological markers were statistically different relative to the control group (Table 4).

The strict adjustment (α = 0.05) in the Bonferroni-adjusted post hoc analysis reduced Type I errors but may have obscured subtle differences (e.g., GGT in NALC: *p* = 0.085). Large mean differences were observed for CD5L: Δ ≈ −9.1.

### 3.4. Analysis and Comparison of Kruskal–Wallis Test Results for Hematological and Enzymatic Parameters

Kruskal–Wallis tests were conducted to evaluate whether the distributions of hematological and enzymatic parameter biomarkers differed significantly across distinct categorical groupings. The null hypothesis (H_0_) was rejected for all parameters at *p* < 0.05, with the exception of age (*p* = 0.183), which showed no significant distributional differences.

**Figure 2:**
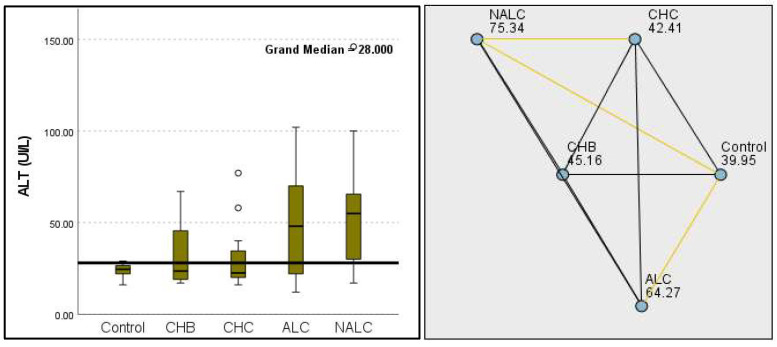
Comparison of the median of alanine aminotransferase (ALT UI/L) levels across control, CHB, CHC, ALC, and NALC groups using Kruskal–Wallis analysis of variance (ANOVA; *p* < 0.05 (**right**)), followed by pairwise-comparison post hoc tests (**left**). The cut-off line is set as the median reference.

**Figure 3:**
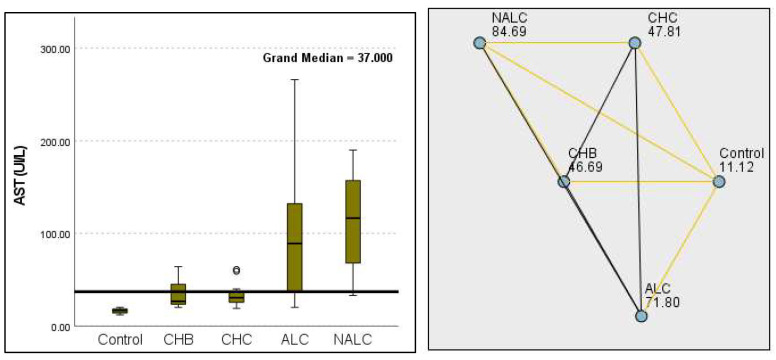
Comparison of median of aspartate aminotransferase (AST UI/L) levels across control, CHB, CHC, ALC, and NALC groups using Kruskal–Wallis analysis of variance (ANOVA; *p* < 0.05 (**right**)), followed by the pairwise-comparison post hoc tests (**left**). The cut-off line is set as the median reference.

**Figure 4:**
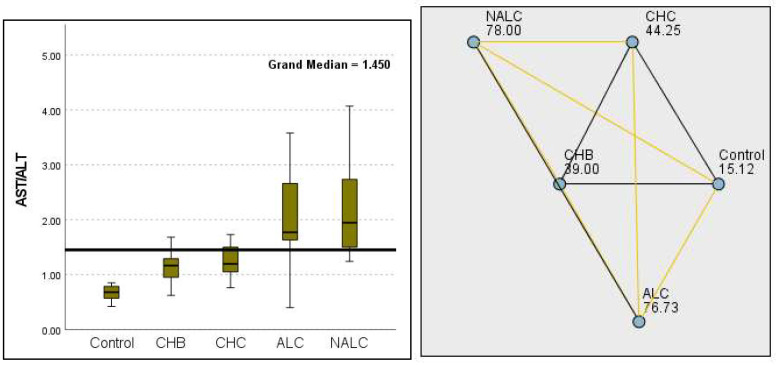
Comparison of the median of AST/ALT ratios across control, CHB, CHC, ALC, and NALC groups using Kruskal–Wallis analysis of variance (ANOVA; *p* < 0.05 (**right**)), followed by pairwise-comparison post hoc tests (**left**). The cut-off line is set as the mean value of the reference range.

Regarding liver enzymes and function markers, a noteworthy distinction in ALT levels was observed among the groups, with a *p*-value of 0.00097 indicating a significant variation. The median ALT values increased progressively from the reference group (39.95 UI/L) to NALC (75.34 UI/L), highlighting a clear upward trend. Similarly, aspartate aminotransferase (AST) levels exhibited a highly significant *p*-value of 2.24 × 10^−13^ (Figure 2). The median AST levels also demonstrated a marked increase, with the control group exhibiting levels between 16.5 and 20 and NALC ranging from 33 to 190 (Figure 3). Notably, the AST/ALT ratio (Figure 4) also exhibited significant differences (*p*= 2.71 × 10^−13^), emphasizing the need for continued investigation into the relationship between these liver function markers and clinical outcomes.

**Figure 5:**
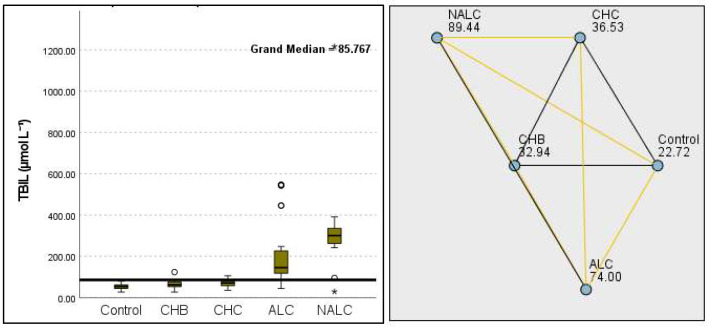
Comparison of the median of TBIL (total bilirubin µmol L^−1^) levels across the control, CHB, CHC, ALC, and NALC groups using Kruskal–Wallis analysis of variance (ANOVA; *p* < 0.05 (**right**)), followed by pairwise-comparison post hoc tests (**left**). The cut-off line is set as the median reference.

The total bilirubin levels were significantly different across the groups, as evidenced by a *p*-value of 8.85 × 10^−14^. The control group exhibited a median of 22.72 µmol L^−1^, while NALC reached 89.44 µmol L^−1^, indicating a concerning increase in bilirubin as group categorization ascended (Figure 5).

**Figure 6:**
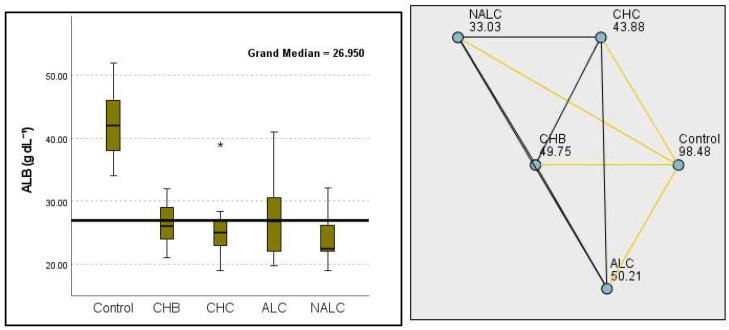
Comparison of the median of ALB (albumin g dL^−1^) levels across control, CHB, CHC, ALC, and NALC groups using Kruskal–Wallis analysis of variance (ANOVA; *p* < 0.05 (**right**)), followed by pairwise-comparison post hoc tests (**left**). The cut-off line is set as the median reference.

This study also revealed significant differences in albumin levels (*p* = 1.87 × 10^−10^), with the control group exhibiting levels notably higher than cohorts with CHB, CHC, and NALC. The median albumin concentration in the control group was approximately 4.2 g/dL, suggesting a potential protective factor against liver dysfunction. Correspondingly, albumin levels exhibited a significant decrease from the control group (98.48 g dL^−1^) to the NALC (33.03 g dL^−1^) groups, reinforcing the trend in diminished hepatic function with an increase in group categorization (Figure 6).

**Figure 7:**
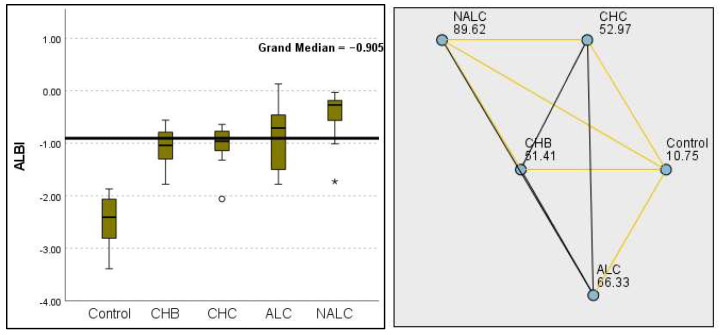
Comparison of the median of ALBI scores across control, CHB, CHC, ALC, and NALC groups using Kruskal–Wallis analysis of variance (ANOVA; *p* < 0.05 (**right**)), followed by pairwise-comparison post hoc tests (**left**). The cut-off line is set as the median reference.

**Figure 8:**
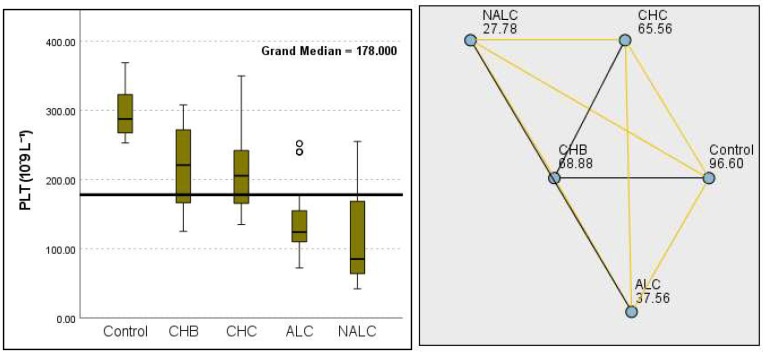
Comparison of the median of PLT (number of platelets in 10^9^ L^−1^) levels across control, CHB, CHC, ALC, and NALC groups using Kruskal–Wallis analysis of variance (ANOVA; *p* < 0.05 (**right**)), followed by pairwise-comparison post hoc tests (**left**). The cut-off line is set as the median reference.

**Figure 9:**
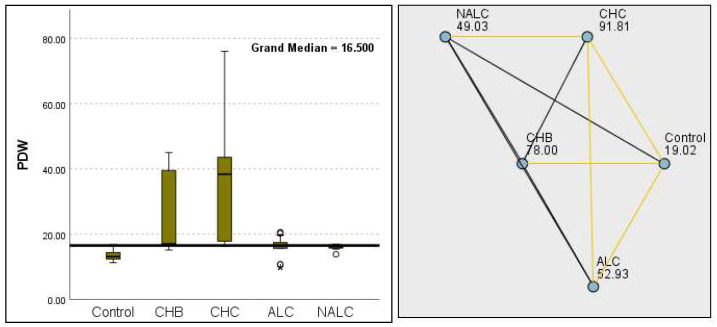
Comparison of the median of final PDW (platelet distribution width) levels (fL) across control, CHB, CHC, ALC, and NALC groups using Kruskal–Wallis analysis of variance (ANOVA; *p* < 0.05 (**right**)), followed by pairwise-comparison post hoc tests (**left**). The cut-off line is set as the median reference.

**Figure 10:**
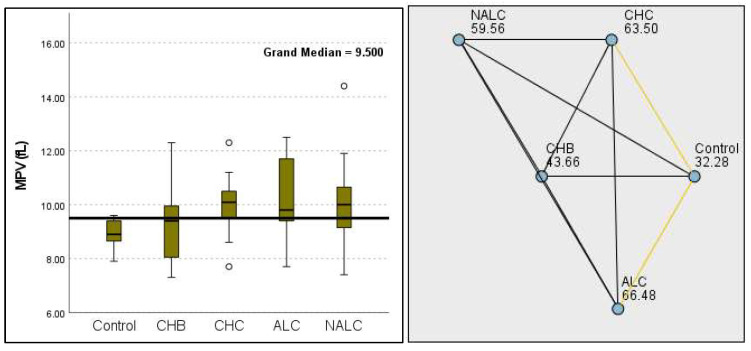
Comparison of the median of MPV (fL) levels across control, CHB, CHC, ALC, and NALC groups using Kruskal–Wallis analysis of variance (ANOVA; *p* < 0.05 (**right**)), followed by pairwise-comparison post hoc tests (**left**). The cut-off line was set as the median reference.

Kruskal–Wallis analysis of variance (ANOVA) also revealed that the ALBI score increased as follows: control > CHB > CHC > ALC > NALC (Figure 7). In contrast, the analysis of PLT revealed significant differences (*p* = 6.78 × 10^−13^), with a decreasing trend from the control (287.5) to NALC (85) groups. This trend suggests a relationship between thrombocytopenia and liver dysfunction, which has considerable implications for patient management and prognostic assessments (Figure 8). In contrast, PDW exhibited levels that were notably higher than CHB and CHC (Figure 9), while MPV exhibited higher levels relative to ALC and CHC (Figure 10).

**Figure 11:**
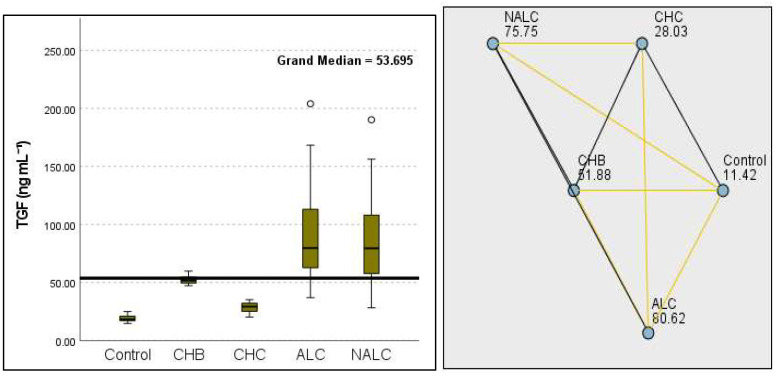
Comparison of the median of TGF β1 (ng mL^−1^) levels across control, CHB, CHC, ALC, and NALC groups using Kruskal–Wallis analysis of variance (ANOVA; *p* < 0.05 (**right**)), followed by pairwise-comparison post hoc tests (**left**). The cut-off line is set as the median reference.

**Figure 12:**
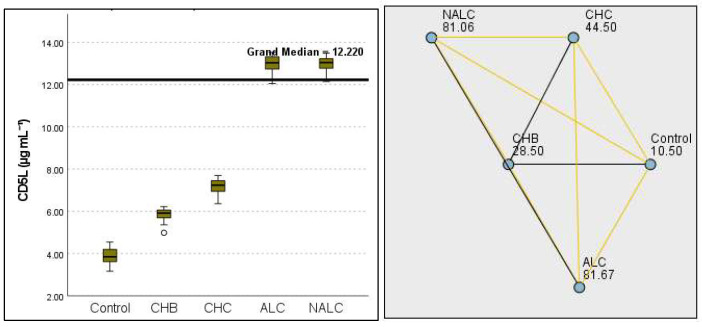
Comparison of the median of CD5L (µg mL^−1^) levels across control, CHB, CHC, ALC, and NALC groups using Kruskal–Wallis analysis of variance (ANOVA; *p* < 0.05 (**right**)), followed by pairwise-comparison post hoc tests (**left**). The cut-off line is set as the median reference.

**Figure 13:**
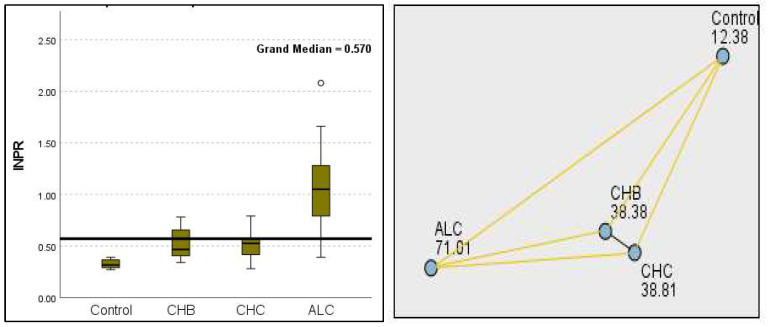
Comparison of the median of INPR levels across control, CHB, CHC, ALC, and NALC groups using Kruskal—Wallis analysis of variance (ANOVA; *p* < 0.05 (**right**)), followed by pairwise-comparison post hoc tests (**left**). The cut-off line is set as the median reference.

**Figure 14:**
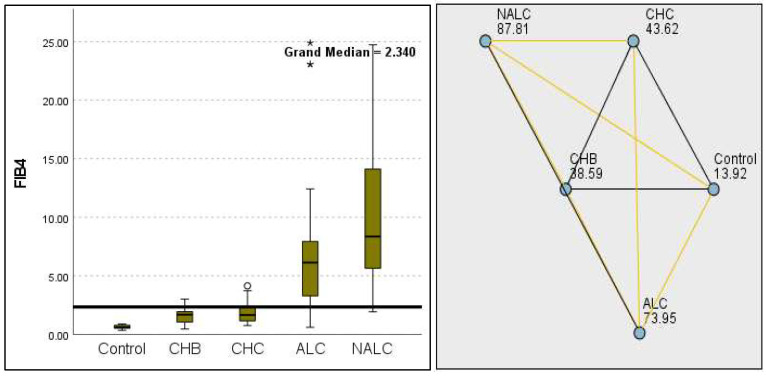
Comparison of median of FIB−4 levels across control, CHB, CHC, ALC, and NALC groups using Kruskal–Wallis analysis of variance (ANOVA; *p* < 0.05 (**right**)), followed by pairwise-comparison post hoc tests (**left**). The cut-off line is set as the median reference.

**Figure 15:**
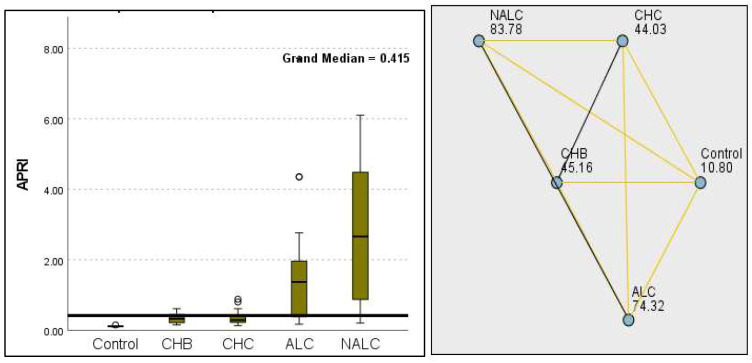
Comparison of the median of APRI levels across control, CHB, CHC, ALC, and NALC groups using Kruskal–Wallis analysis of variance (ANOVA; *p* < 0.05 (**right**)), followed by pairwise-comparison post hoc tests (**left**). The cut-off line is set as the median reference.

**Figure 16:**
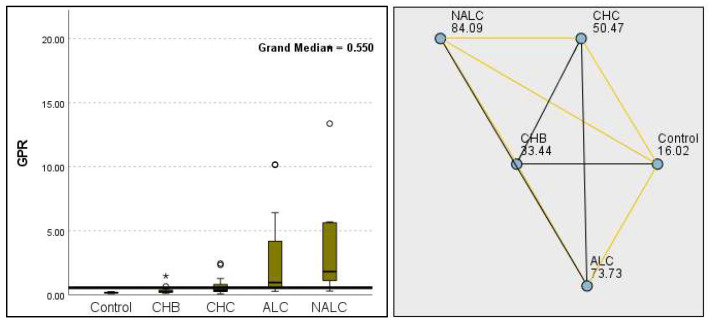
Comparison of the median of GPR levels across control, CHB, CHC, ALC, and NALC groups using Kruskal–Wallis analysis of variance (ANOVA; *p* < 0.05 (**right**)), followed by pairwise-comparison post hoc tests (left). The cut-off line is set as the median reference.

**Figure 17:**
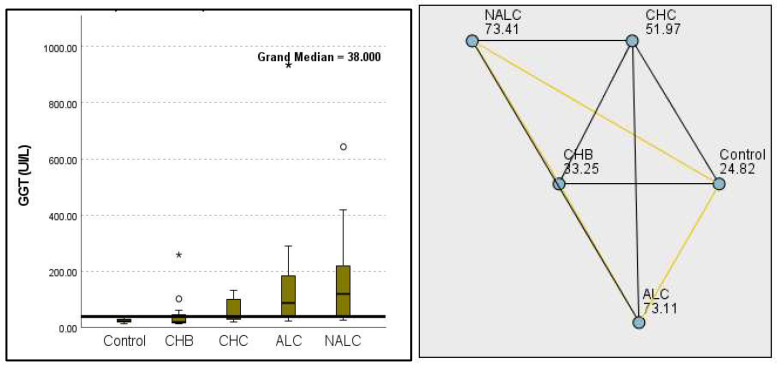
Comparison of the median of gamma-glutamyl transferase (GGT UI/L) levels across control, CHB, CHC, ALC, and NALC groups using Kruskal–Wallis analysis of variance (ANOVA; *p* < 0.05 (**right**)), followed by pairwise-comparison post hoc tests (**left**). The cut-off line is set as the median reference.

**Figure 18:**
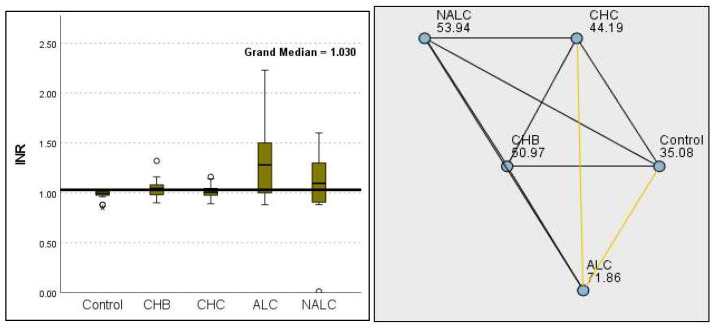
Comparison of the median of INR levels across control, CHB, CHC, ALC, and NALC groups using Kruskal–Wallis analysis of variance (ANOVA; *p* < 0.05 (**right**)), followed by pairwise-comparison post hoc tests ((**left**); *p* < 0.05) The cut-off line is set as the median reference.

The most compelling evidence against H_0_ was observed for TGF (Figure 11) and CD5L (Figure 12), in which the *p*-values suggested near-perfect separation between outcome groups. These biomarkers likely represent critical discriminators in the pathophysiology of the studied outcomes. Comparably robust evidence was found for the following: INPR, *p* = 1.25 × 10^−14^ (Figure 13); FIB4, *p =* 1.29 × 10^−14^ (Figure 14); and APRI *p* = 1.99 × 10^−14^ (Figure 15). All demonstrated *p*-values relative to the order of 10^−14^, which underscores their strong prognostic utility. GPR, *p* = 2.24 × 10^−1^³ (Figure 16), and GGT, *p* = 5.44 × 10^−9^ (Figure 17), followed with highly significant but comparatively weaker results, while INR, *p* = 2.33 × 10^−4^ (Figure 18), exhibited the least robust results, although it was still statistically significant, as evidenced among the cohort.

### 3.5. Analysis of AUC-ROC Results in Different Liver Conditions

The stratification accuracy of each assay was rigorously evaluated through receiver operating characteristic (ROC) curve analysis, which yielded statistically optimized thresholds (cut-off points) with corresponding sensitivity and specificity values (Table S2). As the disease groups were defined using clinical criteria without histologic or elastographic confirmation, the ROC curve analyses reflect discriminatory performance against clinically suspected (rather than gold-standard-verified) diagnoses. Based on the cut-off points, sensitivity, specificity, and 95% CI, the top-performing biomarkers with AUC = 1.000 and perfect discrimination (100% sensitivity and specificity at the established cut-offs) are presented in Table 5. For the sake of brevity, only the most clinically actionable markers are highlighted. Additional markers (e.g., TGF, CD5L) may warrant further validation, as perfect performances may reflect overfitting, and validation in larger cohorts is recommended.

**Figure 19:**
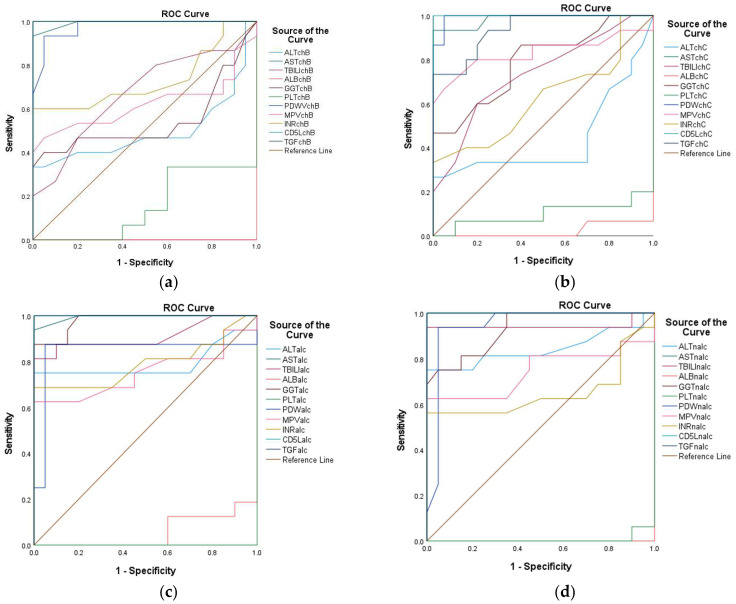
Receiver operating characteristic (ROC) curves of ALT, AST, TBILI, ALB, GGT, PLT, PDW, MPV, INR, CD5L, and TGF-β1 for the prediction of different liver conditions among patients with CHB (**a**), CHC (**b**), ALC (**c**), and NALC (**d**).

Among the evaluated variables, both AST in CHB and NALC exhibited the highest AUC of 1.000 (95% CI: 1.000–1.000), indicating flawless discrimination between the positive and negative groups. In contrast, ALT in CHB exhibited an AUC of 0.527, indicating a limited ability to discriminate effectively between the two states, approaching the threshold of random chance (0.5). For ALT in CHC, while the initial sensitivity was also high, it decreased significantly as the cut-off increased, indicating a trade-off between sensitivity and specificity. In contrast, the ALT in NALC maintained high sensitivity (up to 0.938) while preserving a reasonable specificity rate, rendering it a more reliable test (Figure 19). The *p*-values for the AUC indicate that ALT in NALC is statistically significant (*p* < 0.001), suggesting that its ability to discriminate between positive and negative states is unlikely due to chance. The two other tests did not demonstrate statistically significant discrimination (*p* > 0.05). The paired DeLong test results showed the largest AUC difference of −0.367 (*p* = 0.001) for ALT in NA—suggesting superior discriminative performance over ALT in CHC—and a moderate difference of −0.316 (*p* = 0.039) for ALT in ALC when compared to CHC. No significant differences (*p* = 0.659) were observed for ALT in ALC vs. ALT in NALC, suggesting comparable performances between these two biomarkers.

The AUC of 0.931 for the AST/ALT ratio in CHB indicated excellent discrimination. Sensitivity remains high across multiple thresholds, with a notable decrease in specificity as sensitivity decreases. The AST/ALT ratio in CHC revealed an AUC of 0.967, suggesting slightly better performance compared to CHB, with high sensitivity across nearly all thresholds. For the AST/ALT ratio in ALC, AUC = 0.929, which indicates a strong predictive ability, similarly to CHB. In contrast, for the AST/ALT ratio in NALC, AUC = 1.000, indicating perfect discrimination and implying that it is highly effective in identifying NALC.

The ALBI score exhibited perfect AUC values for CHB, ALC, and NALC, confirming its robustness across different liver diseases; moreover, for CHC, AUC = 0.984, reflecting excellent performance.

The GPR in CHB (AUC = 0.758) indicated moderate discriminatory power, suggesting that it may be less reliable in this context. The GPR in CHC (AUC = 0.897) exhibited a significant improvement. The GPRs in ALC and in NALC both showed AUC values above 0.9, indicating good performance, and they significantly outperformed the GPR in CHB (AUC difference = −0.242, *p* = 0.008 for both). The 95% confidence intervals (CIs: −0.422 to −0.063) excluded zero, suggesting superior discriminative utility. The AUC difference recorded between other pairwise biomarkers was not statistically significant according to the DeLong test.

**Figure 20:**
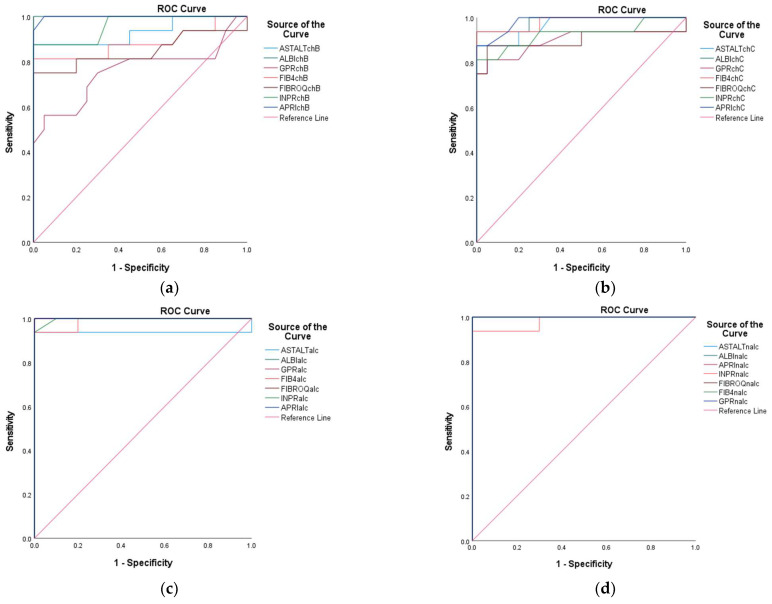
Receiver operating characteristic (ROC) curves of AST/ALT, ALBI, GPR, FIB-4, FIBROQ, INPR, and APRI for the prediction of different liver conditions among patients with CHB (**a**), CHC (**b**), ALC (**c**), and NALC (**d**).

The FIB-4 in CHB showed an AUC = 0.883, suggesting good performance. However, it was not as high as the ALBI score. In contrast, for CHC, the AUC was 0.981, indicating strong discriminative ability. The FIB-4 in ALC reflected AUC = 0.970, which is nearly as effective as the most sensitive tests, while the FIB-4 in NALC had an AUC = 1.000, indicating perfect discrimination (Figure 20).

For FibroQ, statistical analysis indicated an AUC = 0.847 for CHB, showing good potential. Moreover, AUC = 0.900 was observed for CHC, indicating robust performance, and for ALC and NALC, both tests demonstrated strong performance, with values over 0.900 (Figure 20). Near-significant differences were observed for the AUCs of FIBROQ in ALC and NALC versus CHB (AUC difference = −0.153, *p* = 0.054 for both pairs). The 95% confidence intervals (CIs: −0.309 to 0.003) narrowly included zero, suggesting the marginal superiority of FIBROQ in ALC and NALC. According to the DeLong test, the AUC null difference (*p* = 1.000) suggested the identical performance of FIBROQ in ALC and NALC.

The INPR displayed high AUC values across all liver conditions assessed (INPR in CHB: 0.959; INPR in CHC: 0.927; INPR in ALC: 0.999; INPR in NALC: 0.981), indicating its potential as a highly sensitive and specific tool for liver disease diagnosis. The near-perfect AUC in ALC suggests that it could play a critical role in clinical settings, in which distinguishing between different types of liver pathology is necessary (Figure 20).

The statistical analysis of APRI in CHB (0.998) indicates excellent predictive capability. Moreover, the APRI in CHC (0.983) maintained high sensitivity. The APRI in ALC (1.000) indicated perfect discrimination, and the APRI in NALC (1.000) again indicated robustness.

The INPR and APRI scores yield high AUC values, indicating their potential as effective screening tools in various liver disease contexts and reinforcing their role in clinical assessments. The APRI exhibited remarkable consistency, achieving perfect discrimination in both ALC and NALC cases (Figure 20).

The AUC for CD5L was reported at 1.000, indicating that it is a perfect marker for diagnosing the positive state. This performance suggests that CD5L can reliably differentiate individuals with conditions from those without conditions, making it a potentially valuable biomarker for clinical practice. The CD5L in CHB, CHC, ALC, and NALC achieved flawless discrimination with a value of AUC = 1.000 (*p* < 0.001), indicating a complete separation between the positive and negative groups. The 95% CIs [1.000–1.000] and standard errors (0.000) suggest absolute precision relative to these estimates. The TGF β1 marker also displayed an impressive AUC of 0.942, indicating high sensitivity and specificity (Figure 20). Borderline significant differences were revealed using the DeLong test for TGF in CHB compared to TGF in CHC (AUC difference = 0.058, *p* = 0.097; CI: −0.010 to 0.126). These results suggest the marginal superiority of TGF in CHB.

Similarly, the APRI in CHB and NALC demonstrated robust performance, reflecting AUC values of 0.998 and 1.000, respectively. These results reinforce the discriminative performance of the APRI as a predictive variable. Additionally, the performance of the AST/ALT ratio (AUC = 0.931) stands out among the other variables, suggesting that it is a strong candidate for clinical use, particularly when combined with other reliable tests, such as FIB-4 in CHC and AST in NALC (Figure 20).

Most tests with AUC values below 0.6 indicated weak discrimination, and this included ALB in CHB; PLT in CHB and CHC; GPR in CHB; and INR in CHB. The DeLong analysis revealed that the GPR in ALC and NALC significantly outperformed GPR in CHB (AUC difference = −0.242, *p* = 0.008 for both). Moreover, the 95% confidence intervals (CIs: −0.422 to −0.063) excluded zero, suggesting superior discriminative utility. The GPR in ALC and NALC exhibited identical performance (AUC difference = 0.000, *p* = 1.000). However, these low AUC values suggest that these variables may lack meaningful discriminative insights and may require further refinement or re-evaluation.

**Figure 21:**
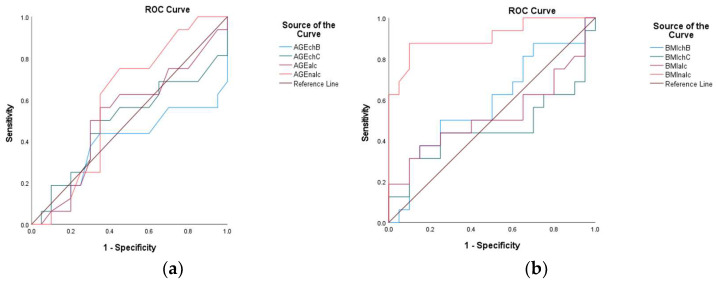
Receiver operating characteristic (ROC) curves of AGE (**a**) and BMI (**b**) for the prediction of different liver conditions among patients with CHB, CHC, ALC, and NALC.

From the analysis of the studied variable, the AUC indicated a low discriminative value for the AGE in CHB and CHC. In contrast, in ALC and NALC, the value was above 0.5. For the BMI variable. The AUC value corresponding to NALC was 0.908, indicating that this parameter exhibits high sensitivity. On the other hand, for CHB, CHC, and ALC, the AUC exhibited values below 0.5 (Figure 21). Significant pairwise differences were observed relative to the BMI in NALC when compared to ALC (AUC difference = −0.384, *p* < 0.001; CI: −0.579 to −0.190; CHB (AUC difference = −0.320, *p* = 0.002; CI: −0.527 to −0.114); and CHC (AUC difference = −0.448, *p* < 0.001; CI: −0.667 to −0.229).

The ROC curve analysis yielded varying AUC values across the examined biomarkers, with values closer to 1.000 indicating excellent test performance. For instance, CD5L and TGF β1 demonstrated outstanding discriminative performance, with high AUC values indicating perfect sensitivity and specificity at various threshold levels. The notably higher AUC values of CD5L and TGFβ1- in comparison with APRI- suggest that while APRI can serve as a valuable screening tool for liver fibrosis, there may be limitations in its specificity compared to more sensitive markers.

Despite numerical differences in AUCs, no biomarker pair exhibited statistically significant differences (*p* > 0.05) for all comparisons; this includes AST, AST/ALT, ALBI, FIB4, INPR, APRI, ALB, INR, and AGE. These phenomena are likely due to the limited sample size, and future studies should validate these results in larger independent cohorts and explore the mechanistic basis of universal performance.

### 3.6. Bivariate Spearman Correlation Analysis Across Liver Cirrhosis Etiologies

The application of bivariate Spearman correlation analyses across CHB, CHC, ALC, and NALC revealed both shared and distinct pathophysiological patterns, underscoring the complex interplay of biomarkers in liver disease progression.

#### 3.6.1. Shared Patterns of Hepatic Injury and Fibrosis

Across all etiologies, the strong positive correlations between ALT and AST (CHB: 0.790**; CHC: 0.827**; ALC: 0.734**; NALC: 0.802**) reflect their universal role as markers of hepatocyte damage (Figures S1–S4). However, the AST/ALT ratio exhibited divergent behaviors, offering etiological insights. In CHB (Figure S1), the ratio was inversely correlated with ALT (−0.555*), contrasting with CHC (Figure S2), where a stronger inverse relationship (−0.652**) suggests ALT dominance even in advanced fibrosis. In ALC (Figure S3), the ratio’s positive correlation with AST (0.721**)—but not ALT (0.180)—was aligned with the classic profile of ALC, in which AST elevation typically surpasses ALT. This divergence highlights how disease-specific mechanisms and viral persistence in CHB/CHC versus direct toxicity in ALC modulate enzyme dynamics.

Cholestatic markers further differentiated these groups. In CHB, TBIL was correlated with AST (0.511*) and GGT (0.576*), which is indicative of concurrent hepatocellular injury and cholestasis. Similarly, in CHC and ALC, GGT demonstrated robust correlations with ALT (CHC: 0.668**; ALC: 0.678**) and AST (CHC: 0.820**; ALC: 0.696**), emphasizing its role in both viral and alcohol-related cholestasis. However, a direct correlation between TBIL and GGT cannot be discussed if bilirubin fractions are not compared. Moreover, GGT may be better correlated with alkaline phosphatase, which is an early marker for cholestasis. Notably, GGT’s near-perfect correlation with the GPR index in ALC (0.945**) suggests its discriminative utility in alcohol-associated fibrosis.

#### 3.6.2. Thrombocytopenia and Fibrosis as a Universal Hallmark

The inverse relationship between PLT and fibrosis indices exhibited a consistent theme across etiologies. In CHB, PLT strongly correlates with INPR (−0.886**) and FibroQ (−0.521*), paralleling the findings relative to CHC (FIB-4: −0.850**; INPR: −0.952**); ALC (APRI: −0.595**; FIB-4: −0.601**); and NALC (Figure S4) (FIB-4: −0.917**). This universality reinforces thrombocytopenia as a surrogate marker of portal hypertension and advanced fibrosis, irrespective of etiology. However, alternative causes may result in a decrease in platelet production, such as alcohol-related bone marrow suppression [30] or following antiviral therapy [31]. Platelet morphology variations suggest etiology-specific perturbations, such as the inverse correlation between the platelet distribution width (PDW) and mean platelet volume (MPV) in CHB (−0.796**) versus their positive correlation in ALC (0.352*). These differences may reflect underlying factors, such as micronutrient deficiencies or splenic sequestration dynamics.

#### 3.6.3. Convergence and Divergence of Fibrosis Indices

Fibrosis indices exhibited high intercorrelations within each etiology (e.g., CHB: FIB-4/FibroQ 0.874**; CHC: 0.794**; ALC: APRI/FIB-4 0.873**; NALC: FIB-4/FibroQ 0.917**), validating their stratification accuracy. The age variable emerged as a critical covariate, particularly in CHB (FIB-4: 0.874**) and CHC (FIB-4: 0.675**; FibroQ: 0.794**). This likely reflects cumulative viral insults over time. In NALC, age’s positive correlation with BMI (0.535*) hints at metabolic syndrome’s compounding role, although BMI’s lack of direct fibrosis correlation suggests nuanced pathways.

#### 3.6.4. Etiology-Specific Biomarker Dynamics

CD5L demonstrates intriguing variability through its positive correlation with FibroQ in CHB (0.565*). This is contrasted with its inverse relationship in CHC (−0.586*) and NALC (−0.524*), suggesting dual roles in fibrogenesis, being pro-fibrotic in viral contexts and protective in metabolic liver disease. Similarly, TGF-β’s paradoxical lack of correlations in CHB and its negative associations in CHC (INR: −0.499*) and NALC (ALT: −0.715**; AST: −0.508*) challenge its canonical pro-fibrotic role. This may reflect stage-dependent effects, in which advanced fibrosis dampens inflammatory signaling or etiological mechanisms overshadow TGF-β’s activity.

Albumin–bilirubin (ALBI) dynamics also exhibited divergence, with a near-perfect inverse correlation between albumin and ALBI in CHB (−0.917**) and NALC (−0.962**), supporting its construct validity. In contrast, in ALC, ALBI’s negative correlation with AST (−0.430**) and AST/ALT (−0.462**) connects hypoalbuminemia directly to hepatocellular dysfunction.

Notable paradoxes include the AST/ALT ratio’s positive correlation with FibroQ in CHB (0.552*), potentially reflecting ALT decline in late-stage cirrhosis. Additionally, TGF-β exhibited inconsistent behavior, possibly due to small sample sizes (*n* = 16 in CHB) or etiological confounders. The predominance of HBV-focused and HCV-focused cohorts limits generalizability to other etiologies, while the cross-sectional design precludes causal inference.

This comparative analysis elucidates core pathways, hepatocellular injury, thrombocytopenia, and fibrogenesis while also highlighting etiological nuances in biomarker interactions. CD5L’s variable role and TGF-β’s paradoxical associations underscore the need for mechanistic studies that disentangle viral, metabolic, and toxic insults. Larger longitudinal cohorts integrating histopathology and viral load data are essential to validate these findings and refine etiology-specific prognostic models.

## 4. Discussion

Liver fibrosis, a precursor to hepatocellular carcinoma, disrupts the hepatic architecture and function through progressive structural alterations, including diminished metabolic exchange; parenchymal replacement by fibrotic tissue; portal hypertension; and subsequent complications, such as coagulopathy, ascites, and encephalopathy. Central to its pathogenesis is the inflammation-driven activation of hepatic stellate cells into myofibroblasts, which drive extracellular matrix deposition. While liver biopsy remains the diagnostic gold standard [32], its invasiveness has spurred interest in noninvasive alternatives, including elastography and serological biomarkers. Due to the invasive nature of this method, alternative noninvasive methods are being researched, such as elastography (FibroScan), which also presents limitations due to the high costs associated with it [33]. In this context, the use of serological (hematological and biochemical) and non-serological (based on mathematical calculation formulas) biomarkers aims at replacing liver biopsies [34].

Sterling et al. [10] emphasized the need to combine noninvasive liver disease assessments with elastography to improve the determination accuracy of significant fibrosis in patients with CHC. For patients with CHB, Sterling et al. [10] recommend the application of noninvasive tests along with elastography to improve diagnostic accuracy [35]. Although there are no specific serological biomarkers for highlighting the degree of liver damage, the potential advantages of their use may include availability and ease of use in routine medical practice [12]. Our study has several limitations: sample-size constraints, single-center design, reference standard gaps, and required validation. Despite these limitations, multicenter approaches and rigorous phenotyping strengthen our study’s internal validity, while the comparative analysis across etiologies provides novel pathobiological insights.

The increased frequency of male patients with chronic hepatitis type B and C could be explained by the fact that estrogens have a protective role against viral infections [36]. In addition, alcohol consumption is more common among men. Moreover, men have a higher risk of infection than women due to their exposure to numerous risk factors. In this context, it is important to highlight that the younger CHB cohort—compared to the ALC group—reflects the potential age-related progression of alcohol-associated liver disease. This study’s power was sufficient for detecting large effects (Cohen’s *d* > 1.5) in preliminary analyses, as evidenced by the significant results (*p* = 0.023, *d* = 1.595). However, the small sample sizes and variability in group sizes (15–49 across disease categories) limit precision, as reflected in the wide confidence intervals. This indicates that while our study had sufficient power for detecting clinically meaningful and large effects, it was underpowered with respect to identifying smaller and subtler associations.

The significant difference between the increased BMI in patients with NALC compared to the normal BMI determined in the other three hepatic disease groups emphasizes the metabolic basis of NALC compared with viral/alcohol-related etiologies.

Unlike other biomarkers, H_0_ was supported for age (*p* = 0.183), with the median ages across categories exhibiting narrow ranges. This suggests demographic homogeneity in the cohort, with age failing to stratify outcomes.

### 4.1. Biomarkers of Hepatocellular Injury and Synthesis

The most commonly used indicators of liver damage degree are aminotransferases ALT (alanine transaminase), AST (aspartate aminotransferase), and hepatocytic enzymes [37]. Moreover, the serum level of ALT is a good indicator that measures the severity of hepatitis [38], and higher serum ALT levels are associated with alcohol consumption [39].

In our study, significant increases in the plasma levels of AST and ALT in CHB, CHC, ALC, and NALC were observed in relation to reference values. In patients with NALC fatty liver disease, Thong and Quynh [38] reported that the ROC curve of serum ALT levels can predict over 32% involvement of hepatocytes in hepatosteatosis without advanced fibrosis (AUC value = 0.602). The higher AUC= 0.842 value in our study indicates the superior discriminative performance of ALT in NALC. Moreover, AST in CHB and NALC is a particularly important parameter that can be used successfully in current medical practice. The increase in the AST plasma levels has been correlated with an increase in the liver damage degree [40].

The AST/ALT ratio, a hallmark of alcoholic liver injury, was elevated in ALC, with an AUC = 0.938, and in NALC, with an AUC = 1.000; this suggests the escalation of enzyme activity, which is contrasted with the lower ratios in CHB and CHC. These findings align with the distinct pathophysiological mechanisms of viral versus metabolic or toxic liver injury. However, in the literature, lower AUC values have been reported for these biomarkers. In patients with CHB, Lai et al. [41] reported the good performance of the AST/ALT ratio relative to predicting liver cirrhosis with an AUC value of 0.654. Amernia et al. [42] showed that an AUC value of 0.720 relative to the AST/ALT ratio can differentiate fibrosis stages in patients with NALC. Johnson et al. [25] used the AST/ALT ratio to develop a scoring system for detecting advanced fibrosis stages in patients with NALC using other variables, such as age, hyperglycemia, BMI, platelet count, and albumin.

Notably, CHB exhibited a strong ALT–AST correlation (0.790**), although AST often exceeded ALT, also reflecting virus-specific injury patterns. In contrast, CHC exhibited comparable ALT and AST elevations, while ALC and NALC exhibited markedly higher levels, which may be due to divergent etiopathogenic mechanisms. Very high ALT and AST values were identified in patients with ALC and NALC.

Increases in the serum values of these parameters were also reported by Ma et al. [40], and these were associated with the degree of liver damage. Intriguingly, age-modulated ALT dynamics in CHB, with younger patients displaying normal ALT levels despite histological abnormalities. In contrast, older individuals (mean age 51.3 ± 14.3) exhibited elevated ALT levels, aligning with age as a risk factor for fibrosis progression [43]. However, these enzymes have not been shown to be sufficiently sensitive markers in the diagnosis of different fibrosis stages [44].

Human serum albumin is the most abundant plasma protein, and it regulates diverse body functions [45]. This protein is predominantly synthesized in hepatocytes such that the plasma level of albumin is considered a biomarker of liver synthesis function and a higher risk of cirrhosis ascites [35]. Although serum albumin levels can also decrease in many other clinical situations (septic states, nephrotic syndrome, systemic inflammatory disorders, etc.), it can still be considered—but not always—a marker of liver disease severity [46]. Under normal conditions, an optimal plasma albumin level inhibits platelet aggregation, promoting vasodilation [47]. In our study, total bilirubin, a product of heme catabolism, exhibited marked elevation in NALC, suggesting cholestatic contributions to fibrosis via collagen deposition [40]. This progressive increase in total bilirubin levels could have crucial implications regarding hepatic functions and metabolic statuses, warranting further scrutiny of the underlying pathophysiological mechanisms involved.

The ALBI index was inversely correlated with albumin (−0.962 in NALC), which effectively stratifies liver dysfunction severity, particularly in compensated cirrhosis [48]. The recorded gradient of the ALBI index reinforces its utility in stratifying liver dysfunction, with cirrhosis cohorts exhibiting significantly impaired albumin production and bilirubin clearance.

Bilirubin is a bile pigment predominantly produced in the spleen due to the degradation of hemes, and it is a hemoglobin component relative to the reticuloendothelial system [46]. The total bilirubin (TBIL) is significantly different between the groups, with hyperbilirubinemia registered in the case of NALC. An increase in the serum value of this marker is associated with accelerated hemolysis or a reduced hepatocyte cell number [49]. However, in order to determine the cause of hyperbilirubinemia (inability to conjugate due to liver damage or biliary excretion defect), it would be necessary to investigate bilirubin fractions. This progressive increase in bilirubin levels could have critical implications with respect to hepatic function and metabolic status, warranting further scrutiny into the underlying pathophysiological mechanisms involved. Abnormal bile acid metabolism can cause the acceleration of collagen deposition and, implicitly, the acceleration of the progression of fibrosis [40].

### 4.2. Coagulation and Oxidative Stress Markers

Thrombocytopenia, a universal feature of advanced fibrosis, is a result of splenic sequestration, reduced thrombopoietin, and portal hypertension [50,51]. In advanced liver fibrosis, portal hypertension causes hypersplenism and, consequently, the splenic sequestration of platelets [46]. In our study, the most pronounced thrombocytopenia—which is indicative of portal hypertension—was observed in the NALC group, aligning with the presence of advanced fibrosis.

Platelet counts were inversely correlated with fibrosis indices (e.g., INPR: −0.886** in CHB), while INR elevations reflected synthetic impairment, corroborating its predictive value for cirrhosis [52,53]. Our results are similar to those reported in previous studies. Ding et al. [27] reported comparable performances of INPR with GPR, APRI, and FIB-4 for assessing significant fibrosis, but significantly better performance was observed for cirrhosis predictions compared to the other three biomarker panels. In the case of significant fibrosis, the AUROC value was 0.74; for advanced fibrosis, it was 0.76; for cirrhosis, it was 0.86. These values demonstrate excellent performance in the prediction of advanced fibrosis and cirrhosis in CHB patients.

Takaki et al. [54] reported that INR is an independent and significant correlated parameter in patients with CHC. Moreover, in patients with liver cirrhosis induced by the C virus, the prothrombin–INR score can be used to predict the occurrence of esophageal varices [55]. The study developed by Kim et al. [53] reported that there is an increase in the INR in liver cirrhosis. Similar results (a decrease in the number of platelets in parallel with an increase in the INR) were reported by Bashour et al. [56] and Wright et al. [57] with respect to patients with liver cirrhosis. In these circumstances, these two biomarkers (number of platelets and INR) can be used as predictors of liver fibrosis.

GGT, which is linked to oxidative stress and metabolic syndrome, increased progressively across etiologies, with ALC and NALC showing comparable elevations; this implicates shared cholestatic and inflammatory pathways [58]. Some studies show that the increased serum level of GGT is associated with an increased risk of developing metabolic syndrome, which manifests at the hepatic level in nonalcoholic fatty liver disease [59,60]. Petta et al. [58] reported that, in individuals with chronic liver diseases, the serum level of GGT can independently predict the onset and evolution of liver fibrosis. The increased serum level of GGT is also associated with obesity, as observed in patients with NALC. Thus, there is a positive correlation between fibrosis and cirrhosis and the plasma level of GGT. The response of hepatocytes to inflammation plays a decisive role in the physiopathology of hepatic fibrosis, which involves the recruitment of both pro-inflammatory and anti-inflammatory cells, such as monocytes and macrophages. The recruitment of macrophages in response to hepatocytic inflammation has a decisive role in the pathophysiology of liver fibrosis [61].

### 4.3. Immune Mediators and Fibrogenic Pathways

CD5L, a macrophage-derived glycoprotein, increased significantly in chronic hepatitis (CHB and CHC) and cirrhosis (ALC and NALC), modulating inflammation and lipid metabolism [13,62,63,64]. Its uniform elevation in ALC and NALC suggests its conserved role in fibrogenesis, contrasting with TGF-β1’s paradoxical behavior. Despite its canonical pro-fibrotic role, TGF-β1’s elevation parallels aminotransferase activities, supporting its dual function in stellate cell survival and hepatocyte apoptosis [65,66]. Thus, the role of TGF-β1 as a predictive biomarker in liver fibrosis aligns with its antiproliferative, proapoptotic, and immunosuppressive activity. The TGF β1 biomarker is particularly interesting due to its role in fibrosis and tissue remodeling. TGF β1 is considered the promoter of protein accumulation in the extracellular matrix because it stimulates hepatic stellate cells. It also induces hepatocyte apoptosis in normal livers [67]. On the other hand, TGF- β1 can inhibit the apoptosis of stellate cells, thus ensuring their survival [65]. Clinical research revealed that TGF-β1 is associated with the impairment degree of liver functions in CHB and CHC [66,68], liver cirrhosis [66], primary biliary cirrhosis [69], and alcoholic cirrhosis [70]. In particular, TGF-β1 and IL17 are considered biomarkers for monitoring liver inflammation and fibrosis in patients with CHC because they are associated with liver degeneration and fibrosis stages. TGF-β1 can also be used in antifibrotic therapy in patients with HCC in order to mitigate disease progression [71,72].

The close resemblance in the performance of TGF β1 relative to CD5L suggests that both biomarkers may be pivotal in similar pathophysiological processes, warranting further investigation into their combined utility in clinical settings. However, the distinct behavior of CD5L and TGF-β1 across etiologies reveals fundamental differences in their pathophysiological roles. CD5L’s consistent elevation across all disease stages underscores its role as a pan-fibrotic mediator, and it has three key implications: macrophage polarization, metabolic reprogramming, and fibrosis threshold effects. In contrast to CD5L’s consistency, TGF-β1 exhibits etiology-specific behavior, which reveals its context-dependent biology, such as viral hepatitis or alcoholic cirrhosis.

### 4.4. Comparative Utility of Noninvasive Indices

Although the AST/ALT ratio, a longstanding cirrhosis predictor, exceeded 1 in CHB and 2 in ALC/NALC, reflecting etiology-specific injury patterns [73]; its limited sensitivity necessitates complementary indices.

In patients with chronic hepatitis C, the value of the AST/ALT ratio is lower compared to the control value due to disease progression. In general, in chronic type B and C viral liver diseases, the value of the AST/ALT ratio is >1.00, with the exception of the acute stage of the disease. The increase in plasma AST levels is a result of mitochondrial extravasation during liver fibrosis, which has a much higher proportion than cytoplasmic ALT in liver cells. In patients with ALC and NALC, the value of this index is greater than 2.00. In this situation, the serum level of AST is substantially higher than ALT, which suggests constant chronic damage inflicted on hepatocytes. Numerous studies have shown that an increase in the value of this ratio could be predictive of cirrhosis [74,75]. An AST/ALT ratio value of >1.00 may suggest the onset of cirrhosis in patients with chronic viral hepatitis and nonalcoholic steatohepatitis [70]; however, this phenomenon alone is not sufficient for predicting significant fibrosis [23]. This ratio is usually greater than 2.00 in alcoholic liver disease and less than 1.00 in patients with chronic hepatitis and chronic cholestatic syndromes [73]. Notably, in end-stage cirrhosis, both enzymes may normalize due to extensive tissue destruction, limiting the ratio’s utility in advanced disease. This continuum mirrors the pathophysiology of chronic liver injury, where fibrosis progression alters transaminase dynamics through mitochondrial disruption and hepatocyte depletion [76].

APRI and FIB-4, which have been validated across various etiologies, exhibited associations with robust diagnostic performance in our cohort, with an AUC value between 0.883 for FIB-4 in CHB; and 1.000 in NALC and APRI in ALC and NALC.

APRI’s progressive increase aligns with fibrosis stages [34], while FIB-4’s age-integrative design effectively stratifies advanced fibrosis [77]. In CHB, statistical analysis indicates the excellent predictive capacity of APRI. It also maintains its sensitivity in ALC and NALC, indicating its potential for use as a biomarker for establishing liver fibrosis and cirrhosis degrees. Thus, an APRI of <0.2 excludes fibrosis, while an APRI of <0.5 excludes cirrhosis. Our data agree with those obtained by Loaeza-Castillo et al. [44] and indicate the usefulness of using this biomarker in current medical practice, thus avoiding the use of liver biopsies. Research carried out by Moosavy et al. [34] also reported the predictive nature of APRI in determining the degree of liver fibrosis in CHB patients. In this context, Sha et al. [77] reported that APRI is a non-invasive biomarker with a much better predictive character for fibrosis compared to FIB-4 and the AST/ALT and AST/ALT/platelet ratios. The WHO has also advised using the APRI for assessing liver fibrosis in CHB, with a threshold of 0.5–1.5 for significant fibrosis [7]. However, it is estimated that the use of several indices in combination with an algorithmic approach could result in increased stratification accuracy compared to the use of APRI alone [78].

Although FIB-4 was only validated in patients with CHC, our findings suggest its potential utility as a biomarker of fibrosis in patients with CHC, ALC, and NALC. According to Shah et al. [77], an FIB-4 value between 0.68 and 1.37 indicates fibrosis stages 0–2, and an FIB-4 value between 1.28 and 3.08 indicates fibrosis stages 3–4, while FIB-4 values above 3.08 indicate liver cirrhosis. The data indicate that patients who have an FIB-4 value lower than 2 are patients (CHB and CHC) with a high degree of liver fibrosis (stage 3–4), while patients with an FIB-4 value above 3 (ALC and NALC) have liver cirrhosis. Therefore, patients who have an FIB-4 value greater than 1.5 and/or an APRI greater than 0.5 should consider undergoing liver elastography [10]. There is also a limitation in the use of FIB-4: it does not differentiate between a fatty liver and steatohepatitis and therefore cannot be used for the diagnosis of nonalcoholic steatohepatitis (NASH) [77].

FibroQ—a noninvasive biomarker introduced by Hsieh et al. [28]—and GPR—an alternative noninvasive index proposed by Lemoine et al. [24] for liver biopsy or Fibroscan—further enhance stratification accuracy. Specifically, FibroQ outperforms APRI in CHC, while GPR exhibits good discriminative performance in ALC/NALC. Lemoine et al. [24] compared GPR with APRI and FIB-4, and they observed that the AUROC of GPR was superior to APRI and FIB-4 in determining significant fibrosis and advanced fibrosis. Moreover, in their meta-analysis, Lian et al. [79] reported that the AUROC values of GPR in predicting significant fibrosis, advanced fibrosis, and cirrhosis were 0.733, 0.777, and 0.796, respectively. Thus, it can be stated that GPR had moderate diagnostic accuracy in predicting fibrosis and cirrhosis related to CHB. On the other hand, Ekin et al. [80] reported that GPR has moderate accuracy in establishing significant and advanced fibrosis, with AUROC values of 0,721 and 0,796, respectively, and high accuracy in determining cirrhosis (AUROC of 0,851) in patients with CHB. Previous studies have shown that FibroQ has a better predictive performance for fibrosis than APRI [28]. Hsieh et al. [28] reported that in patients with HCC, FibroQ has better predictive values for significant fibrosis (METAVIR ≥ 3) and cirrhosis (METAVIR = 4) than APRI, but it is equal to the AST/ALT ratio with AUC values of 0.783 and 0.791, respectively. Higher UC values were obtained relative to our samples (Table S2).

It can also be stated that FibroQ has similar predictive performances compared to the AST/ALT ratio. The study conducted by Hsieh et al. [81] reported FibroQ’s effectiveness in predicting fibrosis in patients with CHC. Our results confirm the robustness of this biomarker in identifying the degree of fibrosis, observing significant differences between the values of this parameter in all four investigated groups (CHB, CHC, ALC, and NALC).

In our study, the FIB-4 and FibroQ scores—while generally showing strong discriminatory power, particularly relative to CHC and ALC—do not reach the same performance levels as the ALBI score and APRI in certain contexts. Their moderate AUC values in chronic hepatitis B suggest that, while they can be useful, they may not be the first-line options for liver disease screening in this patient population.

The wide range of fibrosis indices in cirrhotic groups (e.g., GPR in ALC: 0.27–10.15; FibroQ in NALC: 3.05–46.7) may indicate heterogeneous fibrosis stages or etiological subphenotypes. The strong correlation of all indices with disease progression supports their combined use for noninvasive staging, although outliers (e.g., CHC’s AST/ALT discrepancy) necessitate cautious interpretation and dataset verification.

### 4.5. Strengths and Clinical Implications, Limitations, and Future Directions

This study’s strength lies in its multi-etiology cohort, enabling the comparative analysis of biomarkers across CHB, CHC, ALC, and NALC, and the findings suggest that employing multifactorial approaches in liver pathology may improve diagnosis accuracy. However, external validation is required. By validating noninvasive indices in diverse populations, critical gaps in fibrosis diagnostics can be addressed, particularly in resource-limited settings. The integration of serological (e.g., CD5L, TGF-β1) and mathematical indices (APRI and FIB-4) provides a holistic diagnostic framework, reducing reliance on invasive biopsies. Furthermore, this study emphasizes age and metabolic factors as pivotal modifiers of fibrosis progression, offering insights into personalized risk stratification.

However, several limitations must be acknowledged. The single-center, hospital-based recruitment system may introduce referral bias, limiting the generalizability of our results to broader populations. Our study’s cross-sectional design restricts temporal or causal inferences, only capturing a snapshot of disease status. The absence of a histological or elastographic gold standard means that ROC curve analyses are relative to clinical diagnosis, which may introduce diagnostic misclassifications. Small subgroup sizes (*n* = 15–16) could inflate area under the curve (AUC) estimates, potentially overestimating biomarker performances due to the limited variability and overfitting. Furthermore, potential residual confounding from factors such as metabolic syndrome or antiviral therapy status cannot be fully excluded.

Despite these constraints, our findings advocate for algorithmic approaches that combine elastography and biomarkers to optimize diagnostic precision, offering scalable and noninvasive fibrosis monitoring in clinical practice.

## 5. Conclusions

In this single-center cross-sectional study, several serological biomarkers (AST, ALT, TBIL, ALB, PLT, INR, GGT, CD5L, and TGFβ1) and non-serological indices (AST/ALT ratio, ALBI score, GPR, APRI, FIB-4, INPR, and FibroQ) exhibit moderate-to-high discriminative performance for ≥F3 fibrosis across four etiologies, including CHB, CHC, ALC, and NALC. The findings highlight the prognostic utility of these biomarkers in stratifying liver fibrosis.

This study underscores the potential of multifactorial biomarker panels in bridging pathophysiological mechanisms with clinical outcomes, enabling the earlier detection and etiology-specific management of liver fibrosis. By aligning serological, hematologic, and composite indices with disease mechanisms, clinicians can reduce diagnostic inertia, particularly in resource-limited settings where noninvasive tools are critical.

The causal relationship between biomarker elevation and fibrosis progression remains unestablished, and regional biases (e.g., the Romanian cohort demographics) limit generalizability. Additionally, overlapping metabolic and viral pathways challenge biomarker specificity. Prospective multicenter validation with histological or elastographic endpoints is warranted before clinical adoption. Future studies should prioritize longitudinal designs to establish temporal relationships and refine mathematical models incorporating predictive variables, ultimately reducing reliance on invasive diagnostic methods.

## Figures and Tables

**Table 1:** Reference ranges of serological and non-serological parameter s in adults with chronic liver diseases (CHB, CHC, ALC, and NALC) and healthy controls; comparison against established clinical thresholds for liver fibrosis and dysfunction.

Investigated Parameter	Reference Ranges/Values [Minimum–Maximum]
Serological biomarkers	
AST (UI/L)	[8–48]
ALT (UI/L)	[7–55]
ALB (g dL^−1^)	[35–52]
TBIL (µmol L^−1^)	[1.71–20.52]
GGT (UI/L)	Women: [5–36] Man: [8–61]
PLT (109 L−1)	[150–450]
CD5L (µg mL^−1^)	[3.16–6.06]
TGFβ1 (ng mL^−1^)	[18.28–70.92]
INR	[0.8–1.2]
PDW	[9–17%]
MPV (fL)	[7.2–11.7]
Non-serological biomarkers	
AST/ALT	[0.7–1.4] **>1.0—**hepatic conditions with necrosis **>2.0—**alcoholic hepatitis **>3.0—**ass/ociated with biliary cirrhosis
ALBI	Grade 1 (≤−2.60): mild liver dysfunction Grade 2 (>−2.60 to ≤−1.39): moderate liver dysfunction Grade 3 (>−1.39): severe liver dysfunction
FIB-4	<1.45: no significant fibrosis 1.45–3.25: moderate fibrosis >3.25: significant fibrosis
APRI	<1.0: no significant fibrosis 1.0–2.0: moderate fibrosis >2.0: significant fibrosis
GPR	Cut-off of 0.32: predictive of significant fibrosis Cut-off of 0.56: predictive of cirrhosis
INPR	<0.5: no fibrosis 0.5–1.0: significant fibrosis >1.0: cirrhosis
FibroQ	<0.6: no fibrosis 0.6–1.6: significant fibrosis >2.6: cirrhosis

**Table 2:** Comparative analysis of demographic and clinical characteristics (gender distribution, age, BMI, and etiology) between adults with chronic liver diseases (CHB, CHC, ALC, and NALC) and healthy controls using independent-sample *t*-tests.

Group	Gender (F/M)	Age (Years), [Range]	BMI (kg/m²), [Range]	Etiology
Control	10/10	56.5 ± 10.5 [35–75]	25.0 ± 3.8 [17.3–32.6]	-
CHB	5/11	51.3 ± 14.3 [34–73]	25.5 ± 4.0 [17.4–32.5]	HBV
CHC	6/9	55.87 ± 13.38 [38–78]	24.53 ± 6.1 [16.5–28.4]	HBC
ALC	11/38	57.37 ± 10.08 [40–77]	26.22 ± 6.34 [17.4–38.5]	Alcoholism
NALC	8/8	57.5 ± 7.4 [47–76]	34.98 ± 6.4 * [32.3–43.8]	Steatohepatitis

* Data are expressed as mean ± SD. The mean value columns within each row with the same superscript denote significantly different observations (*p* ≤ 0.05).

**Table 3:** Comparative analysis of direct serum biomarker values among patients with chronic liver diseases (CHB, CHC, ALC, and NALC) and healthy controls using independent-sample *t*-tests.

Direct Serum Biomarker	Mean ± Standard Deviation [Range]				
	CONTROL	CHB	CHC	ALC	NALC
AST (UI/L)	16.2 ± 2.83 [12–20]	33.93 ± 15 * [20–64]	33.62 ± 11.83 * [19–61]	99.85 ± 74.41 * [20–266]	111.75 ± 49.95 * [47–190]
ALT (UI/L)	24.15 ± 3.39 [16–29]	31.37 ± 15.57 [17–67]	29.62 ± 16.66 [16–77]	46.38 ± 25.22 * [16–102]	57.43 ± 34.21 * [17–146]
Albumin (g dL^−1^)	42.35 ± 5.11 * [34–52]	26.31 ± 3.23 * [21–32]	25.57 ± 4.35 * [18.9–39]	27.50 ± 6.03 * [19.7–41]	23.96 ± 4.00 * [22–32.1]
Total Bilirubin (µmol L^−1^)	52.60 ± 15.83 [26.52–79.57]	64.65 ± 24.10 [26.52–123.78]	67.97 ± 18.46 * [35.36–106.1]	199.82 ± 141.03 * [44.21–546.43]	332.01 ± 253.12 * [95.49–715.77]
GGT (UI/L)	23.5 ± 6.89 [12–36]	45.06 ± 61.34 [16–258]	59.33 ± 42.49 * [18–132]	184.81 ± 212.84 * [22–935]	168.5 ± 175.98 * [25–418]
PLT (10^9^ L^−1^)	297.4 ± 35.46 [253–369]	216.75 ± 60.36 * [133–308]	209.46 ± 58.81 * [135–287]	164.18 ± 61.1 * [72–252]	115.06 ± 70.70 * [42–255]
CD5L (µg mL^−1^)	3.86 ± 0.37 [3.16–4.54]	5.81 ± 0.35 * [4.98–6.22]	7.17 ± 0.39 * [6.36–7.69]	12.99 ± 0.37 * [12.24–13.52]	12.98 ± 0.34 * [12.14–13.4]
TGFβ1 (ng mL^−1^)	19.13 ± 2.89 [14.69–24.36]	51.99 ± 3.48 * [47.26–59.78]	28.49 ± 4.84 * [20.14–33.21]	91.08 ± 35.34 * [36.9–139.8]	87.07 ± 42.15 * [28.2–156.3]
INR	0.98 ± 0.05 [0.85–1.03]	1.04 ± 0.09 * [0.96–1.16]	1.01 ± 0.07 [0.91–1.14]	1.25 ± 0.32 * [0.88–2.23]	1.08 ± 0.36 [0.88–1.6]
PDW	13.34 ± 1.49 [11.4–16.8]	26.05 ± 11.72 * [16–45]	36.24 ± 19.12 * [16.3–76.1]	15.91 ± 2.43 * [9.8–20.6]	16.05 ± 0.79 * [13.8–16.9]
MPV	8.95 ± 0.52 [7.9–9.6]	9.22 ± 1.28 [7.3–12.3]	9.98 ± 1.09 * [7.7–12.3]	10.43 ± 1.61 * [8.5–12.3]	9.98 ± 1.69 * [7.4–14.4]

* Data are expressed as mean ± SD. The mean values within a row with the same superscript denote significantly different observations (*p* ≤ 0.05).

**Table 4:** Comparative analysis of indirect non-serological biomarker values among patients with chronic liver diseases (CHB, CHC, ALC, and NALC) and healthy controls using independent-sample *t*-tests.

Indirect Non-Serological Biomarkers	Mean ± Standard Deviation [Range]				
	CONTROL	CHB	CHC	ALC	NALC
AST/ALT ratio	0.67 ± 0.12 [0.42–0.85]	1.14 ± 0.27 * [0.62–1.68]	1.23 ± 0.29 * [0.76–1.73]	2.41 ± 0.91 * [0.4–3.58]	2.14 ± 0.77 * [1.33–4.07]
ALBI score	−2.47 ± 0.44 [−3.39–1.87]	−1.05 ± 0.33 * [−1.78–0.56]	−1.01 ± 0.34 * [−2.06–0.64]	−0.85 ± 0.55 * [−1.78 + 0.13]	−0.43 ± 0.43 * [−1.73–0.03]
GPR	0.16 ± 0.04 [0.1–0.25]	0.34 ± 0.33 * [0.1–1.46]	0.71 ± 0.72 * [0.2–2.44]	2.73 ± 2.69 * [0.27–10.15]	4.15 ± 5.22 * [0.28–19.29]
APRI	0.10 ± 0.02 [0.07–0.15]	0.33 ± 0.13 * [0.15–0.61]	0.36 ± 0.22 * [0.13–0.88]	1.96 ± 2.0 * [0.17–4.35]	2.75 ± 2.09 * [0.2–0.61]
FIB−4	0.62 ± 0.16 [0.34–0.89]	1.58 ± 0.73 * [0.46–3.01]	1.87 ± 0.98 * [0.75–4.14]	7.21 ± 5.51 * [1.4–23.06]	10.63 ± 7.41 * [1.93–24.73]
INPR	0.32 ± 0.04 [0.27–0.39]	0.51 ± 0.15 * [0.34–0.78]	0.51 ± 0.13 * [0.28–0.79]	0.85 ± 0.4 * [0.39–2.08]	1.39 ± 0.79 * [0.35–2.14]
FibroQ	1.31 ± 0.36 [0.77–1.79]	3.25 ± 1.86 * [0.72–7.15]	3.53 ± 2.08 * [0.17–9.23]	12.99 ± 9.51 * [2.26–15.78]	17.31 ± 12.05 * [3.05–46.7]

* Data are expressed as mean ± SD. The mean values within a row with the same superscript are significantly different (*p* ≤ 0.05) relative to the Control group.

**Table 5:** Top-performing serum biomarkers for liver fibrosis stratified via etiology (CHB, CHC, ALC, and NALC) using AUC, cut-off points, sensitivity, specificity, and 95% confidence intervals from ROC curve analysis.

Etiology	Biomarker	AUC	Cut-Off Points	Sensitivity	Specificity
CHB	ALBI	1.000 * (95% CI: 1.000–1.000)	−1.8250	3	100%
	CD5L		4.7600	100%	
	TGF		36.1050		
CHC	CD5L	1.000 * (95% CI: 1.000–1.000)	5.4500	100%	100%
ALC	AST	1.000 * (95% CI: 1.000–1.000)	20.5000	100%	100%
	GPR		0.2600		
	APRI		0.1950		
	FIB4		1.1450		
NALC	AST	1	26.5000	100%	100%
	GPR	1.000 * (95% CI: 1.000–1.000)	0.2650		
	FIB4		1.4100		

* Internal estimate—requires external validation.

## Data Availability

The original contributions presented in this study are included in the article. Further inquiries can be directed to the corresponding authors.

## Supplementary Materials

The following supporting information can be downloaded at: https://www.mdpi.com/article/10.3390/diagnostics15131575/s1, Table S1. Bonferroni Post-Hoc Multiple Comparisons of Biomarker Profiles Among Study Groups (CHB, CHC, ALC, NALC, and Controls); Table S2. Diagnostic performance of biomarkers and non-invasive scores for liver fibrosis stratification: area under the curve (AUC) analysis by etiology (CHB, CHC, ALC, NALC); Figure S1. Heat map based on bivariate Spearman correlations in CHB (degrees of freedom differ, n=14 vs. 48); Figure S2. Heat map based on bivariate Spearman correlations in CHC (degrees of freedom differ, n=14 vs. 48); Figure S3. Heat map based on bivariate Spearman correlations in ALC (degrees of freedom differ, n=14 vs. 48).

## Abbreviations

The following abbreviations are used in this manuscript:

CHB	Chronic hepatitis B
CHC	Chronic hepatitis C
ALC	Alcoholic cirrhosis
NALC	Nonalcoholic cirrhosis
AST	Aspartate aminotransferase
ALT	Alanine aminotransferase
TBIL	Total bilirubin
ALB	Albumin
PLT	Number of platelets
INR	International normalized ratio
GGT	Gamma-glutamyl transferase
CD5L	CD 5 antigen-like
TGFβ1	Transforming growth factor
PDW	Platelet distribution width
MPV	Mean platelet volume
BMI	Body mass index
