# Supplementary material for: Evaluating the Discriminative Performance of Noninvasive Biomarkers in Chronic Hepatitis B/C, Alcoholic Cirrhosis, and Nonalcoholic Cirrhosis: A Comparative Analysis
Source: Diagnostics (Basel). 2025 Jun 20;15(13):1575. doi: 10.3390/diagnostics15131575 (PMC12248557; doi:10.3390/diagnostics15131575)
Supplement: Supplementary file 1 [file diagnostics-15-01575-s001.zip › diagnostics-3694305-supplementary.pdf]

Supplementary Material

Evaluating the Diagnostic Reliability of Noninvasive Biomarkers in Chronic Hepatitis B/C, Alcoholic Cirrhosis, and Non-Alcoholic Cirrhosis: A Comparative Analysis

Table S1. Bonferroni Post-Hoc Multiple Comparisons of Biomarker Profiles Among Study Groups (CHB, CHC, ALC, NALC, and Controls)

Dependent Variable	(I) OUTCOME	(J) OUTCOME	Mean Difference (I-J)	Std. Error	Sig.	95% Confidence Interval Lower Bound	95% Confidence Interval Upper Bound
ALT	Control	CHB	-7.22500	7.43975	1.000	-28.5589	14.1089
		CHC	-5.47500	7.43975	1.000	-26.8089	15.8589
		ALC	-22.23095*	6.02613	.004	-39.5112	-4.9507
		NALC	-33.28750*	7.43975	.000	-54.6214	-11.9536
	CHB	Control	7.22500	7.43975	1.000	-14.1089	28.5589
		CHC	1.75000	7.84219	1.000	-20.7380	24.2380
		ALC	-15.00595	6.51646	.233	-33.6923	3.6804
		NALC	-26.06250*	7.84219	.012	-48.5505	-3.5745
	CHC	Control	5.47500	7.43975	1.000	-15.8589	26.8089
		CHB	-1.75000	7.84219	1.000	-24.2380	20.7380
		ALC	-16.75595	6.51646	.115	-35.4423	1.9304
		NALC	-27.81250*	7.84219	.006	-50.3005	-5.3245
	ALC	Control	22.23095*	6.02613	.004	4.9507	39.5112
		CHB	15.00595	6.51646	.233	-3.6804	33.6923
		CHC	16.75595	6.51646	.115	-1.9304	35.4423
		NALC	-11.05655	6.51646	.927	-29.7429	7.6298
	NALC	Control	33.28750*	7.43975	.000	11.9536	54.6214

		CHB	26.06250*	7.84219	.012	3.5745	48.5505
		CHC	27.81250*	7.84219	.006	5.3245	50.3005
		ALC	11.05655	6.51646	.927	-7.6298	29.7429
AST	Control	CHB	-17.73750	17.01073	1.000	-66.5168	31.0418
		CHC	-17.42500	17.01073	1.000	-66.2043	31.3543
		ALC	-83.65714*	13.77853	.000	-123.1679	-44.1463
		NALC	-95.55000*	17.01073	.000	-144.3293	-46.7707
	CHB	Control	17.73750	17.01073	1.000	-31.0418	66.5168
		CHC	.31250	17.93089	1.000	-51.1054	51.7304
		ALC	-65.91964*	14.89965	.000	-108.6453	-23.1940
		NALC	-77.81250*	17.93089	.000	-129.2304	-26.3946
	CHC	Control	17.42500	17.01073	1.000	-31.3543	66.2043
		CHB	-.31250	17.93089	1.000	-51.7304	51.1054
		ALC	-66.23214*	14.89965	.000	-108.9578	-23.5065
		NALC	-78.12500*	17.93089	.000	-129.5429	-26.7071
	ALC	Control	83.65714*	13.77853	.000	44.1463	123.1679
		CHB	65.91964*	14.89965	.000	23.1940	108.6453
		CHC	66.23214*	14.89965	.000	23.5065	108.9578
		NALC	-11.89286	14.89965	1.000	-54.6185	30.8328
	NALC	Control	95.55000*	17.01073	.000	46.7707	144.3293
		CHB	77.81250*	17.93089	.000	26.3946	129.2304
		CHC	78.12500*	17.93089	.000	26.7071	129.5429
		ALC	11.89286	14.89965	1.000	-30.8328	54.6185
ASTALT	Control	CHB	-.46587	.20522	.252	-1.0544	.1226
		CHC	-.55588	.20522	.079	-1.1444	.0326
		ALC	-1.39305*	.16623	.000	-1.8697	-.9164
		NALC	-1.47213*	.20522	.000	-2.0606	-.8836
	CHB	Control	.46587	.20522	.252	-.1226	1.0544
		CHC	-.09000	.21632	1.000	-.7103	.5303
		ALC	-.92717*	.17975	.000	-1.4426	-.4117
		NALC	-1.00625*	.21632	.000	-1.6266	-.3859

	CHC	Control	.55588	.20522	.079	-.0326	1.1444
		CHB	.09000	.21632	1.000	-.5303	.7103
		ALC	-.83717*	.17975	.000	-1.3526	-.3217
		NALC	-.91625*	.21632	.000	-1.5366	-.2959
	ALC	Control	1.39305*	.16623	.000	.9164	1.8697
		CHB	.92717*	.17975	.000	.4117	1.4426
		CHC	.83717*	.17975	.000	.3217	1.3526
		NALC	-.07908	.17975	1.000	-.5945	.4364
	NALC	Control	1.47213*	.20522	.000	.8836	2.0606
		CHB	1.00625*	.21632	.000	.3859	1.6266
		CHC	.91625*	.21632	.000	.2959	1.5366
		ALC	.07908	.17975	1.000	-.4364	.5945
TBILI	Control	CHB	-12.04722	43.85668	1.000	-137.8090	113.7145
		CHC	-15.36297	43.85668	1.000	-141.1247	110.3988
		ALC	-147.21786*	35.52349	.001	-249.0837	-45.3520
		NALC	-279.40679*	43.85668	.000	-405.1685	-153.6450
	CHB	Control	12.04722	43.85668	1.000	-113.7145	137.8090
		CHC	-3.31575	46.22900	1.000	-135.8803	129.2488
		ALC	-135.17064*	38.41394	.006	-245.3250	-25.0163
		NALC	-267.35956*	46.22900	.000	-399.9241	-134.7950
	CHC	Control	15.36297	43.85668	1.000	-110.3988	141.1247
		CHB	3.31575	46.22900	1.000	-129.2488	135.8803
		ALC	-131.85489*	38.41394	.009	-242.0092	-21.7005
		NALC	-264.04381*	46.22900	.000	-396.6083	-131.4793
	ALC	Control	147.21786*	35.52349	.001	45.3520	249.0837
		CHB	135.17064*	38.41394	.006	25.0163	245.3250
		CHC	131.85489*	38.41394	.009	21.7005	242.0092
		NALC	-132.18893*	38.41394	.008	-242.3433	-22.0346
	NALC	Control	279.40679*	43.85668	.000	153.6450	405.1685
		CHB	267.35956*	46.22900	.000	134.7950	399.9241
		CHC	264.04381*	46.22900	.000	131.4793	396.6083

		ALC	132.18893*	38.41394	.008	22.0346	242.3433
ALB	Control	CHB	16.03750*	1.69148	.000	11.1871	20.8879
		CHC	16.77500*	1.69148	.000	11.9246	21.6254
		ALC	14.84524*	1.37009	.000	10.9164	18.7740
		NALC	18.38750*	1.69148	.000	13.5371	23.2379
	CHB	Control	-16.03750*	1.69148	.000	-20.8879	-11.1871
		CHC	.73750	1.78298	1.000	-4.3753	5.8503
		ALC	-1.19226	1.48157	1.000	-5.4407	3.0562
		NALC	2.35000	1.78298	1.000	-2.7628	7.4628
	CHC	Control	-16.77500*	1.69148	.000	-21.6254	-11.9246
		CHB	-.73750	1.78298	1.000	-5.8503	4.3753
		ALC	-1.92976	1.48157	1.000	-6.1782	2.3187
		NALC	1.61250	1.78298	1.000	-3.5003	6.7253
	ALC	Control	-14.84524*	1.37009	.000	-18.7740	-10.9164
		CHB	1.19226	1.48157	1.000	-3.0562	5.4407
		CHC	1.92976	1.48157	1.000	-2.3187	6.1782
		NALC	3.54226	1.48157	.186	-.7062	7.7907
	NALC	Control	-18.38750*	1.69148	.000	-23.2379	-13.5371
		CHB	-2.35000	1.78298	1.000	-7.4628	2.7628
		CHC	-1.61250	1.78298	1.000	-6.7253	3.5003
		ALC	-3.54226	1.48157	.186	-7.7907	.7062
ALBI	Control	CHB	-1.41700*	.15630	.000	-1.8652	-.9688
		CHC	-1.45887*	.15630	.000	-1.9071	-1.0107
		ALC	-1.61271*	.12660	.000	-1.9758	-1.2497
		NALC	-2.04012*	.15630	.000	-2.4883	-1.5919
	CHB	Control	1.41700*	.15630	.000	.9688	1.8652
		CHC	-.04188	.16476	1.000	-.5143	.4306
		ALC	-.19571	.13690	1.000	-.5883	.1969
		NALC	-.62312*	.16476	.003	-1.0956	-.1507
	CHC	Control	1.45888*	.15630	.000	1.0107	1.9071
		CHB	.04188	.16476	1.000	-.4306	.5143

		ALC	-.15384	.13690	1.000	-.5464	.2387
		NALC	-.58125*	.16476	.006	-1.0537	-.1088
	ALC	Control	1.61271*	.12660	.000	1.2497	1.9758
		CHB	.19571	.13690	1.000	-.1969	.5883
		CHC	.15384	.13690	1.000	-.2387	.5464
		NALC	-.42741*	.13690	.023	-.8200	-.0348
	NALC	Control	2.04012*	.15630	.000	1.5919	2.4883
		CHB	.62312*	.16476	.003	.1507	1.0956
		CHC	.58125*	.16476	.006	.1088	1.0537
		ALC	.42741*	.13690	.023	.0348	.8200
GGT	Control	CHB	-21.56250	54.05562	1.000	-176.5704	133.4454
		CHC	-35.83333	54.05562	1.000	-190.8412	119.1745
		ALC	-145.69048*	43.78454	.012	-271.2454	-20.1356
		NALC	-145.00000	54.05562	.085	-300.0079	10.0079
	CHB	Control	21.56250	54.05562	1.000	-133.4454	176.5704
		CHC	-14.27083	56.97963	1.000	-177.6635	149.1218
		ALC	-124.12798	47.34717	.100	-259.8989	11.6430
		NALC	-123.43750	56.97963	.325	-286.8301	39.9551
	CHC	Control	35.83333	54.05562	1.000	-119.1745	190.8412
		CHB	14.27083	56.97963	1.000	-149.1218	177.6635
		ALC	-109.85714	47.34717	.223	-245.6281	25.9138
		NALC	-109.16667	56.97963	.581	-272.5593	54.2260
	ALC	Control	145.69048*	43.78454	.012	20.1356	271.2454
		CHB	124.12798	47.34717	.100	-11.6430	259.8989
		CHC	109.85714	47.34717	.223	-25.9138	245.6281
		NALC	.69048	47.34717	1.000	-135.0805	136.4614
	NALC	Control	145.00000	54.05562	.085	-10.0079	300.0079
		CHB	123.43750	56.97963	.325	-39.9551	286.8301
		CHC	109.16667	56.97963	.581	-54.2260	272.5593
		ALC	-.69048	47.34717	1.000	-136.4614	135.0805
PLT	Control	CHB	80.65000*	18.55739	.000	27.4355	133.8645

		CHC	87.93333*	18.55739	.000	34.7189	141.1478
		ALC	156.44762*	15.03131	.000	113.3444	199.5508
		NALC	182.33750*	18.55739	.000	129.1230	235.5520
	CHB	Control	-80.65000*	18.55739	.000	-133.8645	-27.4355
		CHC	7.28333	19.56121	1.000	-48.8096	63.3763
		ALC	75.79762*	16.25437	.000	29.1872	122.4080
		NALC	101.68750*	19.56121	.000	45.5945	157.7805
	CHC	Control	-87.93333*	18.55739	.000	-141.1478	-34.7189
		CHB	-7.28333	19.56121	1.000	-63.3763	48.8096
		ALC	68.51429*	16.25437	.001	21.9039	115.1247
		NALC	94.40417*	19.56121	.000	38.3112	150.4971
	ALC	Control	-156.44762*	15.03131	.000	-199.5508	-113.3444
		CHB	-75.79762*	16.25437	.000	-122.4080	-29.1872
		CHC	-68.51429*	16.25437	.001	-115.1247	-21.9039
		NALC	25.88988	16.25437	1.000	-20.7205	72.5003
	NALC	Control	-182.33750*	18.55739	.000	-235.5520	-129.1230
		CHB	-101.68750*	19.56121	.000	-157.7805	-45.5945
		CHC	-94.40417*	19.56121	.000	-150.4971	-38.3112
		ALC	-25.88988	16.25437	1.000	-72.5003	20.7205
GPR	Control	CHB	-.17888	.88145	1.000	-2.7065	2.3487
		CHC	-.54767	.88145	1.000	-3.0753	1.9799
		ALC	-2.18867*	.71396	.028	-4.2360	-.1413
		NALC	-3.99075*	.88145	.000	-6.5184	-1.4631
	CHB	Control	.17888	.88145	1.000	-2.3487	2.7065
		CHC	-.36879	.92913	1.000	-3.0331	2.2955
		ALC	-2.00979	.77206	.106	-4.2237	.2041
		NALC	-3.81187*	.92913	.001	-6.4762	-1.1475
	CHC	Control	.54767	.88145	1.000	-1.9799	3.0753
		CHB	.36879	.92913	1.000	-2.2955	3.0331
		ALC	-1.64100	.77206	.359	-3.8549	.5729
		NALC	-3.44308*	.92913	.003	-6.1074	-.7788

	ALC	Control	2.18867*	.71396	.028	.1413	4.2360
		CHB	2.00979	.77206	.106	-.2041	4.2237
		CHC	1.64100	.77206	.359	-.5729	3.8549
		NALC	-1.80208	.77206	.215	-4.0160	.4118
	NALC	Control	3.99075*	.88145	.000	1.4631	6.5184
		CHB	3.81187*	.92913	.001	1.1475	6.4762
		CHC	3.44308*	.92913	.003	.7788	6.1074
		ALC	1.80208	.77206	.215	-.4118	4.0160
APRI	Control	CHB	-.22475	.48419	1.000	-1.6132	1.1637
		CHC	-.25287	.48419	1.000	-1.6413	1.1356
		ALC	-1.71481*	.39219	.000	-2.8394	-.5902
		NALC	-2.64850*	.48419	.000	-4.0369	-1.2601
	CHB	Control	.22475	.48419	1.000	-1.1637	1.6132
		CHC	-.02812	.51038	1.000	-1.4917	1.4354
		ALC	-1.49006*	.42410	.007	-2.7062	-.2739
		NALC	-2.42375*	.51038	.000	-3.8873	-.9602
	CHC	Control	.25287	.48419	1.000	-1.1356	1.6413
		CHB	.02812	.51038	1.000	-1.4354	1.4917
		ALC	-1.46193*	.42410	.008	-2.6781	-.2458
		NALC	-2.39563*	.51038	.000	-3.8592	-.9321
	ALC	Control	1.71481*	.39219	.000	.5902	2.8394
		CHB	1.49006*	.42410	.007	.2739	2.7062
		CHC	1.46193*	.42410	.008	.2458	2.6781
		NALC	-.93369	.42410	.299	-2.1498	.2824
	NALC	Control	2.64850*	.48419	.000	1.2601	4.0369
		CHB	2.42375*	.51038	.000	.9602	3.8873
		CHC	2.39563*	.51038	.000	.9321	3.8592
		ALC	.93369	.42410	.299	-.2824	2.1498
PDW	Control	CHB	-12.70500*	2.91078	.000	-21.0518	-4.3582
		CHC	-22.89500*	2.91078	.000	-31.2418	-14.5482
		ALC	-2.64071	2.35770	1.000	-9.4016	4.1201

		NALC	-2.71125	2.91078	1.000	-11.0581	5.6356
	CHB	Control	12.70500*	2.91078	.000	4.3582	21.0518
		CHC	-10.19000*	3.06823	.012	-18.9883	-1.3917
		ALC	10.06429*	2.54954	.001	2.7533	17.3753
		NALC	9.99375*	3.06823	.015	1.1954	18.7921
	CHC	Control	22.89500*	2.91078	.000	14.5482	31.2418
		CHB	10.19000*	3.06823	.012	1.3917	18.9883
		ALC	20.25429*	2.54954	.000	12.9433	27.5653
		NALC	20.18375*	3.06823	.000	11.3854	28.9821
	ALC	Control	2.64071	2.35770	1.000	-4.1201	9.4016
		CHB	-10.06429*	2.54954	.001	-17.3753	-2.7533
		CHC	-20.25429*	2.54954	.000	-27.5653	-12.9433
		NALC	-.07054	2.54954	1.000	-7.3815	7.2404
	NALC	Control	2.71125	2.91078	1.000	-5.6356	11.0581
		CHB	-9.99375*	3.06823	.015	-18.7921	-1.1954
		CHC	-20.18375*	3.06823	.000	-28.9821	-11.3854
		ALC	.07054	2.54954	1.000	-7.2404	7.3815
VTM	Control	CHB	-.27000	.43297	1.000	-1.5116	.9716
		CHC	-1.02500	.43297	.197	-2.2666	.2166
		ALC	-1.36405*	.35070	.002	-2.3697	-.3584
		NALC	-1.03250	.43297	.189	-2.2741	.2091
	CHB	Control	.27000	.43297	1.000	-.9716	1.5116
		CHC	-.75500	.45639	1.000	-2.0637	.5537
		ALC	-1.09405*	.37924	.048	-2.1815	-.0066
		NALC	-.76250	.45639	.978	-2.0712	.5462
	CHC	Control	1.02500	.43297	.197	-.2166	2.2666
		CHB	.75500	.45639	1.000	-.5537	2.0637
		ALC	-.33905	.37924	1.000	-1.4265	.7484
		NALC	-.00750	.45639	1.000	-1.3162	1.3012
	ALC	Control	1.36405*	.35070	.002	.3584	2.3697
		CHB	1.09405*	.37924	.048	.0066	2.1815

		CHC	.33905	.37924	1.000	-.7484	1.4265
		NALC	.33155	.37924	1.000	-.7559	1.4190
	NALC	Control	1.03250	.43297	.189	-.2091	2.2741
		CHB	.76250	.45639	.978	-.5462	2.0712
		CHC	.00750	.45639	1.000	-1.3012	1.3162
		ALC	-.33155	.37924	1.000	-1.4190	.7559
INR	Control	CHB	-.06788	.09285	1.000	-.3341	.1984
		CHC	-.03183	.09285	1.000	-.2981	.2344
		ALC	-.35850*	.07521	.000	-.5742	-.1428
		NALC	-.09975	.09285	1.000	-.3660	.1665
	CHB	Control	.06788	.09285	1.000	-.1984	.3341
		CHC	.03604	.09787	1.000	-.2446	.3167
		ALC	-.29063*	.08133	.005	-.5238	-.0574
		NALC	-.03188	.09787	1.000	-.3125	.2488
	CHC	Control	.03183	.09285	1.000	-.2344	.2981
		CHB	-.03604	.09787	1.000	-.3167	.2446
		ALC	-.32667*	.08133	.001	-.5599	-.0935
		NALC	-.06792	.09787	1.000	-.3486	.2127
	ALC	Control	.35850*	.07521	.000	.1428	.5742
		CHB	.29063*	.08133	.005	.0574	.5238
		CHC	.32667*	.08133	.001	.0935	.5599
		NALC	.25875*	.08133	.019	.0255	.4920
	NALC	Control	.09975	.09285	1.000	-.1665	.3660
		CHB	.03188	.09787	1.000	-.2488	.3125
		CHC	.06792	.09787	1.000	-.2127	.3486
		ALC	-.25875*	.08133	.019	-.4920	-.0255
CD5L	Control	CHB	-1.94767*	.12473	.000	-2.3053	-1.5900
		CHC	-3.30900*	.12473	.000	-3.6667	-2.9513
		ALC	-9.11348*	.10103	.000	-9.4032	-8.8238
		NALC	-9.11550*	.12473	.000	-9.4732	-8.7578
	CHB	Control	1.94767*	.12473	.000	1.5900	2.3053

		CHC	-1.36133*	.13148	.000	-1.7384	-.9843
		ALC	-7.16581*	.10925	.000	-7.4791	-6.8525
		NALC	-7.16783*	.13148	.000	-7.5449	-6.7908
	CHC	Control	3.30900*	.12473	.000	2.9513	3.6667
		CHB	1.36133*	.13148	.000	.9843	1.7384
		ALC	-5.80448*	.10925	.000	-6.1178	-5.4912
		NALC	-5.80650*	.13148	.000	-6.1835	-5.4295
	ALC	Control	9.11348*	.10103	.000	8.8238	9.4032
		CHB	7.16581*	.10925	.000	6.8525	7.4791
		CHC	5.80448*	.10925	.000	5.4912	6.1178
		NALC	-.00202	.10925	1.000	-.3153	.3113
	NALC	Control	9.11550*	.12473	.000	8.7578	9.4732
		CHB	7.16783*	.13148	.000	6.7908	7.5449
		CHC	5.80650*	.13148	.000	5.4295	6.1835
		ALC	.00202	.10925	1.000	-.3113	.3153
TGF	Control	CHB	-32.86017*	9.47528	.008	-60.0311	-5.6892
		CHC	-9.36483	9.47528	1.000	-36.5358	17.8061
		ALC	-71.85121*	7.67489	.000	-93.8594	-49.8430
		NALC	-67.94050*	9.47528	.000	-95.1115	-40.7695
	CHB	Control	32.86017*	9.47528	.008	5.6892	60.0311
		CHC	23.49533	9.98782	.205	-5.1454	52.1360
		ALC	-38.99105*	8.29937	.000	-62.7900	-15.1921
		NALC	-35.08033*	9.98782	.007	-63.7210	-6.4396
	CHC	Control	9.36483	9.47528	1.000	-17.8061	36.5358
		CHB	-23.49533	9.98782	.205	-52.1360	5.1454
		ALC	-62.48638*	8.29937	.000	-86.2853	-38.6874
		NALC	-58.57567*	9.98782	.000	-87.2164	-29.9350
	ALC	Control	71.85121*	7.67489	.000	49.8430	93.8594
		CHB	38.99105*	8.29937	.000	15.1921	62.7900
		CHC	62.48638*	8.29937	.000	38.6874	86.2853
		NALC	3.91071	8.29937	1.000	-19.8882	27.7097

	NALC	Control	67.94050*	9.47528	.000	40.7695	95.1115
		CHB	35.08033*	9.98782	.007	6.4396	63.7210
		CHC	58.57567*	9.98782	.000	29.9350	87.2164
		ALC	-3.91071	8.29937	1.000	-27.7097	19.8882
AGE	Control	CHB	5.18750	3.71051	1.000	-5.4526	15.8276
		CHC	.62500	3.71051	1.000	-10.0151	11.2651
		ALC	-3.66667	3.00548	1.000	-12.2851	4.9517
		NALC	-1.00000	3.71051	1.000	-11.6401	9.6401
	CHB	Control	-5.18750	3.71051	1.000	-15.8276	5.4526
		CHC	-4.56250	3.91122	1.000	-15.7782	6.6532
		ALC	-8.85417	3.25002	.075	-18.1738	.4655
		NALC	-6.18750	3.91122	1.000	-17.4032	5.0282
	CHC	Control	-.62500	3.71051	1.000	-11.2651	10.0151
		CHB	4.56250	3.91122	1.000	-6.6532	15.7782
		ALC	-4.29167	3.25002	1.000	-13.6113	5.0280
		NALC	-1.62500	3.91122	1.000	-12.8407	9.5907
	ALC	Control	3.66667	3.00548	1.000	-4.9517	12.2851
		CHB	8.85417	3.25002	.075	-.4655	18.1738
		CHC	4.29167	3.25002	1.000	-5.0280	13.6113
		NALC	2.66667	3.25002	1.000	-6.6530	11.9863
	NALC	Control	1.00000	3.71051	1.000	-9.6401	11.6401
		CHB	6.18750	3.91122	1.000	-5.0282	17.4032
		CHC	1.62500	3.91122	1.000	-9.5907	12.8407
		ALC	-2.66667	3.25002	1.000	-11.9863	6.6530
FIB4	Control	CHB	-.95512	1.50855	1.000	-5.2810	3.3707
		CHC	-1.25012	1.50855	1.000	-5.5760	3.0757
		ALC	-6.27117*	1.22191	.000	-9.7751	-2.7673
		NALC	-10.00575*	1.50855	.000	-14.3316	-5.6799
	CHB	Control	.95513	1.50855	1.000	-3.3707	5.2810
		CHC	-.29500	1.59015	1.000	-4.8549	4.2649
		ALC	-5.31604*	1.32133	.001	-9.1050	-1.5270

		NALC	-9.05063*	1.59015	.000	-13.6105	-4.4908
	CHC	Control	1.25013	1.50855	1.000	-3.0757	5.5760
		CHB	.29500	1.59015	1.000	-4.2649	4.8549
		ALC	-5.02104*	1.32133	.002	-8.8100	-1.2320
		NALC	-8.75563*	1.59015	.000	-13.3155	-4.1958
	ALC	Control	6.27117*	1.22191	.000	2.7673	9.7751
		CHB	5.31604*	1.32133	.001	1.5270	9.1050
		CHC	5.02104*	1.32133	.002	1.2320	8.8100
		NALC	-3.73458	1.32133	.056	-7.5236	.0544
	NALC	Control	10.00575*	1.50855	.000	5.6799	14.3316
		CHB	9.05063*	1.59015	.000	4.4908	13.6105
		CHC	8.75563*	1.59015	.000	4.1958	13.3155
		ALC	3.73458	1.32133	.056	-.0544	7.5236
IMC	Control	CHB	-.46525	1.79534	1.000	-5.6135	4.6830
		CHC	1.26933	1.79534	1.000	-3.8789	6.4176
		ALC	.37600	1.45421	1.000	-3.7940	4.5460
		NALC	-9.94525*	1.79534	.000	-15.0935	-4.7970
	CHB	Control	.46525	1.79534	1.000	-4.6830	5.6135
		CHC	1.73458	1.89246	1.000	-3.6921	7.1613
		ALC	.84125	1.57253	1.000	-3.6681	5.3506
		NALC	-9.48000*	1.89246	.000	-14.9067	-4.0533
	CHC	Control	-1.26933	1.79534	1.000	-6.4176	3.8789
		CHB	-1.73458	1.89246	1.000	-7.1613	3.6921
		ALC	-.89333	1.57253	1.000	-5.4027	3.6160
		NALC	-11.21458*	1.89246	.000	-16.6413	-5.7879
	ALC	Control	-.37600	1.45421	1.000	-4.5460	3.7940
		CHB	-.84125	1.57253	1.000	-5.3506	3.6681
		CHC	.89333	1.57253	1.000	-3.6160	5.4027
		NALC	-10.32125*	1.57253	.000	-14.8306	-5.8119
	NALC	Control	9.94525*	1.79534	.000	4.7970	15.0935
		CHB	9.48000*	1.89246	.000	4.0533	14.9067

		CHC	11.21458*	1.89246	.000	5.7879	16.6413
		ALC	10.32125*	1.57253	.000	5.8119	14.8306

*The mean difference is significant at the 0.05 level.

Table S2. Diagnostic performance of biomarkers and non-invasive scores for liver fibrosis stratification: area under the curve (AUC) analysis by etiology (CHB, CHC, ALC, NALC).

Area Under the Curve

Test Result Variable(s)	Area	Std. Error[a]	Asymptotic Sig.[b]	Asymptotic 95% Confidence Interval	
				Lower Bound	Upper Bound
ALTchB	.495	.114	.960	.272	.718
ASTchB	.993	.009	.000	.975	1.000
TBILIchB	.672	.096	.086	.483	.860
ALBchB	.000	.000	.000	.000	.000
GGTchB	.543	.113	.665	.322	.764
PLTchB	.153	.068	.001	.020	.287
PDWVchB	.977	.020	.000	.937	1.000
MPVchB	.622	.111	.224	.405	.839
INRchB	.732	.096	.021	.543	.921
CD5LchB	1.000	.000	.000	1.000	1.000
TGFchB	1.000	.000	.000	1.000	1.000
ALTchC	.440	.111	.549	.223	.657
ASTchC	.985	.017	.000	.952	1.000
TBILIchC	.735	.087	.019	.564	.906
ALBchC	.022	.023	.000	.000	.067
GGTchC	.790	.079	.004	.636	.944
PLTchC	.100	.066	.000	.000	.229

PDWchC	.993	.009	.000	.976	1.000
MPVchC	.832	.083	.001	.669	.994
INRchC	.625	.101	.211	.427	.823
CD5LchC	1.000	.000	.000	1.000	1.000
TGFchC	.938	.038	.000	.865	1.000
ALTalc	.791	.093	.003	.608	.974
ASTalc	.994	.009	.000	.977	1.000
TBILIalc	.909	.058	.000	.795	1.000
ALBalc	.056	.038	.000	.000	.131
GGTalc	.980	.018	.000	.944	1.000
PLTalc	.000	.000	.000	.000	.000
PDWalc	.844	.086	.000	.676	1.000
MPValc	.753	.093	.010	.571	.935
INRalc	.792	.086	.003	.623	.961
CD5Lalc	1.000	.000	.000	1.000	1.000
TGFalc	1.000	.000	.000	1.000	1.000
ALTnalc	.842	.078	.000	.688	.996
ASTnalc	1.000	.000	.000	1.000	1.000
TBILInalc	.944	.055	.000	.836	1.000
ALBnalc	.000	.000	.000	.000	.000
GGTnalc	.930	.040	.000	.851	1.000
PLTnalc	.006	.008	.000	.000	.023
PDWnalc	.945	.043	.000	.862	1.000
MPVnalc	.744	.096	.013	.556	.932
INRnalc	.650	.107	.126	.440	.860
CD5Lnalc	1.000	.000	.000	1.000	1.000
TGFnalc	1.000	.000	.000	1.000	1.000

ASTALTchB	.931	.049	.000	.836	1.000
ALBIchB	1.000	.000	.000	1.000	1.000
GPRchB	.758	.090	.009	.582	.934
FIB4chB	.883	.067	.000	.751	1.000
FIBROQchB	.847	.078	.000	.695	.999
INPRchB	.959	.031	.000	.899	1.000
APRIchB	.998	.003	.000	.992	1.000
ASTALTchC	.967	.026	.000	.916	1.000
ALBIchC	.984	.017	.000	.950	1.000
GPRchC	.897	.065	.000	.769	1.000
FIB4chC	.981	.020	.000	.942	1.000
FIBROQchC	.900	.067	.000	.769	1.000
INPRchC	.927	.051	.000	.826	1.000
APRIchC	.983	.016	.000	.951	1.000
ASTALTalc	.938	.061	.000	.819	1.000
ALBIalc	1.000	.000	.000	1.000	1.000
GPRalc	1.000	.000	.000	1.000	1.000
FIB4alc	.988	.014	.000	.959	1.000
FIBROQalc	1.000	.000	.000	1.000	1.000
INPRalc	.997	.005	.000	.987	1.000
APRIalc	1.000	.000	.000	1.000	1.000
ASTALTnalc	1.000	.000	.000	1.000	1.000
ALBInalc	1.000	.000	.000	1.000	1.000
APRInalc	1.000	.000	.000	1.000	1.000
INPRnalc	.981	.020	.000	.942	1.000
FIBROQnalc	1.000	.000	.000	1.000	1.000
FIB4nalc	1.000	.000	.000	1.000	1.000

GPRnalc	1.000	.000	.000	1.000	1.000

The test result variable(s): INPRalc has at least one tie between the positive actual state group and the negative actual state group. Statistics may be biased.

[a]Under the nonparametric assumption

[b]Null hypothesis: true area = 0.5

Correlations Spearman's rho	ALTchB	ASTchB	ASTALTchB	TBILIchB	ALBchB	ALBIchB	GGTchB	PLTchB	GPRchB	APRIchB	PDWVchB	MPVchB	INRchB	CD5LchB	TGFchB	INPRchB	AGEchB	FIB4chB	BMIchB	FIBROQchB
**ALTchB**	1	.790**	-.555*	0.308	-0.101	0.241	.666**	0.252	.527*	.500*	-0.182	-0.437	0.487	-0.129	0.08	-0.195	-0.352	-0.201	-0.069	-0.432
**ASTchB**	.790**	1	0.001	.511*	-0.158	0.336	.701**	0.239	0.476	.702**	-0.409	-.678**	0.446	0.187	0.13	-0.132	-0.019	0.116	-0.173	-0.08
**ASTALTchB**	-.555*	0.001	1	0.042	0.041	-0.032	-0.337	-0.075	-0.337	0.134	-0.316	-0.287	-0.262	0.399	0.031	0.069	0.45	0.442	-0.134	.552*
**TBILIchB**	0.308	.511*	0.042	1	-0.298	.617*	.576*	0.059	0.451	0.407	-0.043	-0.189	0.132	0.001	0.101	-0.048	-0.227	0.001	-0.082	-0.088
**ALBchB**	-0.101	-0.158	0.041	-0.298	1	-.917**	-0.287	-0.073	-0.179	0.063	0.086	0.198	-0.159	0.267	0.201	0.101	0.028	0.158	-0.299	0.14
**ALBIchB**	0.241	0.336	-0.032	.617*	-.917**	1	0.453	0.107	0.32	0.11	-0.052	-0.258	0.201	-0.159	-0.09	-0.113	-0.161	-0.175	0.162	-0.175
**GGTchB**	.666**	.701**	-0.337	.576*	-0.287	0.453	1	0.469	.712**	0.249	-0.123	-0.374	.523*	0.039	0.065	-0.357	-0.262	-0.282	0.128	-0.333
**PLTchB**	0.252	0.239	-0.075	0.059	-0.073	0.107	0.469	1	-0.072	-0.432	0.05	-0.385	0.411	-0.165	0.291	-.886**	-0.313	-.594*	-0.256	-.521*
**GPRchB**	.527*	0.476	-0.337	0.451	-0.179	0.32	.712**	-0.072	1	0.494	0.08	0.006	0.413	0.237	0.001	0.115	-0.133	0.068	.498*	-0.05
**APRIchB**	.500*	.702**	0.134	0.407	0.063	0.11	0.249	-0.432	0.494	1	-0.242	-0.238	0.083	0.38	-0.024	0.45	0.165	.562*	0.069	0.297
**PDWVchB**	-0.182	-0.409	-0.316	-0.043	0.086	-0.052	-0.123	0.05	0.08	-0.242	1	.796**	-0.098	0.158	0.285	-0.054	-0.32	-0.202	0.049	-0.252
**MPVchB**	-0.437	-.678**	-0.287	-0.189	0.198	-0.258	-0.374	-0.385	0.006	-0.238	.796**	1	-0.322	0.077	0.076	0.322	-0.139	-0.012	0.281	-0.003
**INRchB**	0.487	0.446	-0.262	0.132	-0.159	0.201	.523*	0.411	0.413	0.083	-0.098	-0.322	1	0.258	0.062	-0.033	0.279	0.068	0.013	0.145
**CD5LchB**	-0.129	0.187	0.399	0.001	0.267	-0.159	0.039	-0.165	0.237	0.38	0.158	0.077	0.258	1	0.299	0.399	0.378	0.432	-0.119	.565*
**TGFchB**	0.08	0.13	0.031	0.101	0.201	-0.09	0.065	0.291	0.001	-0.024	0.285	0.076	0.062	0.299	1	-0.206	-0.23	-0.321	-0.321	-0.215
**INPRchB**	-0.195	-0.132	0.069	-0.048	0.101	-0.113	-0.357	-.886**	0.115	0.45	-0.054	0.322	-0.033	0.399	-0.206	1	.567*	.715**	0.215	.742**
**AGEchB**	-0.352	-0.019	0.45	-0.227	0.028	-0.161	-0.262	-0.313	-0.133	0.165	-0.32	-0.139	0.279	0.378	-0.23	.567*	1	.811**	0.103	.891**
**FIB4chB**	-0.201	0.116	0.442	0.001	0.158	-0.175	-0.282	-.594*	0.068	.562*	-0.202	-0.012	0.068	0.432	-0.321	.715**	.811**	1	0.135	.874**
**BMIchB**	-0.069	-0.173	-0.134	-0.082	-0.299	0.162	0.128	-0.256	.498*	0.069	0.049	0.281	0.013	-0.119	-0.321	0.215	0.103	0.135	1	0.125
**FIBROQchB**	-0.432	-0.08	.552*	-0.088	0.14	-0.175	-0.333	-.521*	-0.05	0.297	-0.252	-0.003	0.145	.565*	-0.215	.742**	.891**	.874**	0.125	1

Figure S1. Heat map based on bivariate Spearman correlations in CHB (degrees of freedom differ, n=14 vs. 48). ** Correlation is significant at the 0.01 level (2- tailed). * Correlation is significant at the 0.05 level (2-tailed).

Correlations Spearman's rho	ALTchC	ASTchC	ASTALTchC	TBILIchC	ALBchC	ALBIchC	GGTchC	PLTchC	GPRchC	APRIchC	PDWchC	MPVchC	INRchC	CD5LchC	TGFchC	INPRchC	AGEchC	FIB4chC	BMIchC	FIBROQchC
**ALTchC**	1	.827**	-.652**	-0.161	0.387	-0.162	.668**	-0.476	.626**	.612*	.564*	.525*	-0.084	.603*	0.137	0.292	0.086	0.321	-0.123	-0.004
**ASTchC**	.827**	1	-0.183	-0.014	0.279	0.057	.820**	-.781**	.799**	.832**	.721**	.606*	-0.041	0.343	0.255	.662**	0.161	.675**	0.009	0.432
**ASTALTchC**	-.652**	-0.183	1	0.227	-0.213	0.366	-0.027	-0.142	-0.032	-0.061	-0.153	-0.34	0.086	-.586*	0.196	0.249	0.052	0.27	0.187	0.463
**TBILIchC**	-0.161	-0.014	0.227	1	0.241	0.256	-0.058	-0.353	0.033	0.277	-0.018	-0.294	-0.09	0.009	-0.404	0.478	-0.045	0.292	0.246	0.456
**ALBchC**	0.387	0.279	-0.213	0.241	1	-.687**	0.301	-0.118	0.187	0.249	-0.132	-0.166	0.019	0.265	-0.154	0.156	0.325	0.269	-0.189	0.186
**ALBIchC**	-0.162	0.057	0.366	0.256	-.687**	1	-0.029	-0.293	0.098	0.158	0.312	0.019	-0.177	-0.039	0.105	0.248	-0.391	0.082	0.415	0.084
**GGTchC**	.668**	.820**	-0.027	-0.058	0.301	-0.029	1	-.664**	.924**	.746**	.626*	0.456	-0.039	0.032	0.343	.626*	0.243	.625*	-0.05	0.321
**PLTchC**	-0.476	-.781**	-0.142	-0.353	-0.118	-0.293	-.664**	1	-.752**	-.941**	-.561*	-0.438	0.165	0.005	-0.136	-.952**	-0.109	-.850**	-0.339	-.696**
**GPRchC**	.626**	.799**	-0.032	0.033	0.187	0.098	.924**	-.752**	1	.725**	.690**	.516*	-0.151	-0.036	0.287	.623**	-0.006	.542*	0.191	0.286
**APRIchC**	.612*	.832**	-0.061	0.277	0.249	0.158	.746**	-.941**	.725**	1	.690**	.583*	-0.287	0.235	0.232	.892**	0.233	.827**	0.081	.530*
**PDWchC**	.564*	.721**	-0.153	-0.018	-0.132	0.312	.626*	-.561*	.690**	.690**	1	.809**	-0.281	0.158	0.389	.518*	-0.251	0.3	0.109	0.066
**MPVchC**	.525*	.606*	-0.34	-0.294	-0.166	0.019	0.456	-0.438	.516*	.583*	.809**	1	-0.279	0.188	0.276	0.394	0.005	0.299	-0.047	0.072
**INRchC**	-0.084	-0.041	0.086	-0.09	0.019	-0.177	-0.039	0.165	-0.151	-0.287	-0.281	-0.279	1	-0.129	-.499*	-0.043	-0.123	-0.127	0.177	0.139
**CD5LchC**	.603*	0.343	-.586*	0.009	0.265	-0.039	0.032	0.005	-0.036	0.235	0.158	0.188	-0.129	1	-0.199	0.026	-0.011	0.068	-0.204	-0.252
**TGFchC**	0.137	0.255	0.196	-0.404	-0.154	0.105	0.343	-0.136	0.287	0.232	0.389	0.276	-.499*	-0.199	1	-0.01	0.193	0.118	-0.385	-0.091
**INPRchC**	0.292	.662**	0.249	0.478	0.156	0.248	.626*	-.952**	.623**	.892**	.518*	0.394	-0.043	0.026	-0.01	1	0.173	.871**	0.287	.741**
**AGEchC**	0.086	0.161	0.052	-0.045	0.325	-0.391	0.243	-0.109	-0.006	0.233	-0.251	0.005	-0.123	-0.011	0.193	0.173	1	.564*	-.753**	0.396
**FIB4chC**	0.321	.675**	0.27	0.292	0.269	0.082	.625*	-.850**	.542*	.827**	0.3	0.299	-0.127	0.068	0.118	.871**	.564*	1	-0.05	.794**
**BMIchC**	-0.123	0.009	0.187	0.246	-0.189	0.415	-0.05	-0.339	0.191	0.081	0.109	-0.047	0.177	-0.204	-0.385	0.287	-.753**	-0.05	1	0.112
**FIBROQchC**	-0.004	0.432	0.463	0.456	0.186	0.084	0.321	-.696**	0.286	.530*	0.066	0.072	0.139	-0.252	-0.091	.741**	0.396	.794**	0.112	1

Figure S2. Heat map based on bivariate Spearman correlations in CHC (degrees of freedom differ, n=14 vs. 48). ** Correlation is significant at the 0.01 level (2- tailed). * Correlation is significant at the 0.05 level (2-tailed).

Correlations Spearman's rho	ALTalc	ASTalc	ASTALTalc	TBILIalc	ALBalc	ALBIalc	GGTalc	PLTalc	GPRalc	APRIalc	PDWalc	MPValc	INRalc	CD5Lalc	TGFalc	INPRalc	AGEalc	FIB4alc	BMIalc	FIBROQalc
**ALTalc**	1	.734**	0.18	.341*	-0.22	0.175	.678**	-.352*	.665**	.776**	0.056	.397**	0.059	0.081	-0.003	0.201	-0.092	.653**	0.031	0.196
**ASTalc**	.734**	1	.721**	0.269	-.430**	.334*	.696**	-.379*	.769**	.919**	-0.013	0.159	-0.166	0.072	-0.158	0.142	-0.238	.845**	0.249	.551**
**ASTALTalc**	0.18	.721**	1	-0.07	-.462**	0.24	.442**	-0.082	.497**	.568**	-0.051	-0.1	-0.235	-0.05	-.307*	-0.107	-.322*	.635**	0.296	.625**
**TBILIalc**	.341*	0.269	-0.07	1	-0.061	.368*	0.167	0.071	0.161	0.174	-0.106	0.219	0.207	.344*	-0.118	0.001	0.011	0.067	0.254	-0.027
**ALBalc**	-0.22	-.430**	-.462**	-0.061	1	-.516**	-.453**	-0.212	-.325*	-0.244	0.227	.312*	-.335*	0.232	0.007	0.041	0.033	-0.213	0.025	-0.204
**ALBIalc**	0.175	.334*	0.24	.368*	-.516**	1	0.262	-0.026	0.256	0.228	0.047	-0.206	0.191	0.163	-0.181	0.086	-0.11	0.099	0.139	0.133
**GGTalc**	.678**	.696**	.442**	0.167	-.453**	0.262	1	0.024	.945**	.585**	-0.027	0.166	-0.019	-0.052	-0.03	-0.168	-0.222	.530**	-0.03	0.155
**PLTalc**	-.352*	-.379*	-0.082	0.071	-0.212	-0.026	0.024	1	-0.202	-.595**	-.334*	-.397**	.305*	-0.058	-0.176	-.693**	-0.019	-.601**	0.051	-.499**
**GPRalc**	.665**	.769**	.497**	0.161	-.325*	0.256	.945**	-0.202	1	.708**	0.071	0.297	-0.206	-0.024	-0.051	-0.074	-0.275	.657**	0.016	0.266
**APRIalc**	.776**	.919**	.568**	0.174	-0.244	0.228	.585**	-.595**	.708**	1	0.128	.324*	-0.258	0.112	-0.094	0.283	-0.22	.873**	0.224	.523**
**PDWalc**	0.056	-0.013	-0.051	-0.106	0.227	0.047	-0.027	-.334*	0.071	0.128	1	.352*	-0.095	0.12	0.002	0.196	-0.05	0.081	0.081	0.131
**MPValc**	.397**	0.159	-0.1	0.219	.312*	-0.206	0.166	-.397**	0.297	.324*	.352*	1	-0.196	0.099	0.004	0.175	-0.215	0.243	-0.117	0.171
**INRalc**	0.059	-0.166	-0.235	0.207	-.335*	0.191	-0.019	.305*	-0.206	-0.258	-0.095	-0.196	1	-0.145	0.168	.390*	0.249	-0.256	0.007	0.094
**CD5Lalc**	0.081	0.072	-0.05	.344*	0.232	0.163	-0.052	-0.058	-0.024	0.112	0.12	0.099	-0.145	1	-0.104	-0.006	-0.052	0.029	.448*	-0.069
**TGFalc**	-0.003	-0.158	-.307*	-0.118	0.007	-0.181	-0.03	-0.176	-0.051	-0.094	0.002	0.004	0.168	-0.104	1	.329*	0.286	-0.047	-0.035	0.098
**INPRalc**	0.201	0.142	-0.107	0.001	0.041	0.086	-0.168	-.693**	-0.074	0.283	0.196	0.175	.390*	-0.006	.329*	1	0.198	0.295	-0.05	.605**
**AGEalc**	-0.092	-0.238	-.322*	0.011	0.033	-0.11	-0.222	-0.019	-0.275	-0.22	-0.05	-0.215	0.249	-0.052	0.286	0.198	1	-0.032	-0.211	-0.216
**FIB4alc**	.653**	.845**	.635**	0.067	-0.213	0.099	.530**	-.601**	.657**	.873**	0.081	0.243	-0.256	0.029	-0.047	0.295	-0.032	1	0.123	.596**
**BMIalc**	0.031	0.249	0.296	0.254	0.025	0.139	-0.03	0.051	0.016	0.224	0.081	-0.117	0.007	.448*	-0.035	-0.05	-0.211	0.123	1	0.194
**FIBROQalc**	0.196	.551**	.625**	-0.027	-0.204	0.133	0.155	-.499**	0.266	.523**	0.131	0.171	0.094	-0.069	0.098	.605**	-0.216	.596**	0.194	1

Figure S3. Heat map based on bivariate Spearman correlations in ALC (degrees of freedom differ, n=14 vs. 48). ** Correlation is significant at the 0.01 level (2- tailed). * Correlation is significant at the 0.05 level (2-tailed).

Correlations Spearman's rho	ALTnalc	ASTnalc	ASTALTnalc	TBILInalc	ALBnalc	ALBInalc	GGTnalc	PLTnalc	GPRnalc	APRInalc	PDWnalc	MPVnalc	INRnalc	CD5Lnalc	TGFnalc	INPRnalc	AGEnalc	FIB4nalc	BMInalc	FIBROQnalc
**ALTnalc**	1	.802**	-0.427	0.111	-.557*	.610*	0.247	-0.069	0.256	0.273	-0.098	0.275	.500*	-0.166	-.715**	0.237	-0.064	0.355	-0.384	0.168
**ASTnalc**	.802**	1	0.117	0.083	-.500*	.516*	.657**	-0.108	.663**	0.371	-0.238	0.407	0.479	-0.406	-.508*	0.246	0.308	.567*	-0.11	0.357
**ASTALTnalc**	-0.427	0.117	1	0.031	0.006	-0.022	0.436	-0.164	.530*	0.173	-0.143	0.118	0.1	-0.437	0.408	0.155	0.296	0.367	0.39	0.44
**TBILInalc**	0.111	0.083	0.031	1	0.019	0.152	0.056	-0.412	0.251	0.307	-0.249	-0.277	-0.052	0.248	0.406	0.314	-0.063	0.251	0.372	0.214
**ALBnalc**	-.557*	-.500*	0.006	0.019	1	-.962**	-0.041	-0.006	-0.124	-0.355	-0.253	-0.107	-0.436	0.106	0.362	-0.191	0.059	-0.23	0.263	-0.205
**ALBInalc**	.610*	.516*	-0.022	0.152	-.962**	1	-0.043	-0.155	0.155	0.468	0.262	0.041	.556*	-0.132	-0.335	0.358	-0.15	0.346	-0.246	0.339
**GGTnalc**	0.247	.657**	0.436	0.056	-0.041	-0.043	1	0.058	.793**	0.134	-.590*	0.421	-0.054	-0.287	0.016	-0.078	0.406	0.352	0.057	0.153
**PLTnalc**	-0.069	-0.108	-0.164	-0.412	-0.006	-0.155	0.058	1	-.498*	-.855**	-0.194	0.062	-0.241	0.262	-0.261	-.921**	0.261	-.815**	0.142	-.858**
**GPRnalc**	0.256	.663**	.530*	0.251	-0.124	0.155	.793**	-.498*	1	.640**	-0.377	0.273	0.222	-0.443	0.172	0.479	0.293	.812**	0.007	.673**
**APRInalc**	0.273	0.371	0.173	0.307	-0.355	0.468	0.134	-.855**	.640**	1	0.207	-0.01	0.325	-0.288	0.205	.865**	-0.231	.853**	-0.271	.829**
**PDWnalc**	-0.098	-0.238	-0.143	-0.249	-0.253	0.262	-.590*	-0.194	-0.377	0.207	1	0.181	0.168	-0.247	-0.022	0.174	-0.199	0.001	-0.186	0.12
**MPVnalc**	0.275	0.407	0.118	-0.277	-0.107	0.041	0.421	0.062	0.273	-0.01	0.181	1	0.295	-.692**	-0.223	-0.049	0.177	0.193	-0.29	0.146
**INRnalc**	.500*	0.479	0.1	-0.052	-0.436	.556*	-0.054	-0.241	0.222	0.325	0.168	0.295	1	-0.422	-0.414	.515*	-0.104	0.452	-0.374	.565*
**CD5Lnalc**	-0.166	-0.406	-0.437	0.248	0.106	-0.132	-0.287	0.262	-0.443	-0.288	-0.247	-.692**	-0.422	1	0.104	-0.269	-0.125	-.577*	0.097	-.524*
**TGFnalc**	-.715**	-.508*	0.408	0.406	0.362	-0.335	0.016	-0.261	0.172	0.205	-0.022	-0.223	-0.414	0.104	1	0.079	-0.025	0.035	0.319	0.115
**INPRnalc**	0.237	0.246	0.155	0.314	-0.191	0.358	-0.078	-.921**	0.479	.865**	0.174	-0.049	.515*	-0.269	0.079	1	-0.323	.838**	-0.29	.898**
**AGEnalc**	-0.064	0.308	0.296	-0.063	0.059	-0.15	0.406	0.261	0.293	-0.231	-0.199	0.177	-0.104	-0.125	-0.025	-0.323	1	0.019	.535*	-0.118
**FIB4nalc**	0.355	.567*	0.367	0.251	-0.23	0.346	0.352	-.815**	.812**	.853**	0.001	0.193	0.452	-.577*	0.035	.838**	0.019	1	-0.147	.917**
**BMInalc**	-0.384	-0.11	0.39	0.372	0.263	-0.246	0.057	0.142	0.007	-0.271	-0.186	-0.29	-0.374	0.097	0.319	-0.29	.535*	-0.147	1	-0.25
**FIBROQnalc**	0.168	0.357	0.44	0.214	-0.205	0.339	0.153	-.858**	.673**	.829**	0.12	0.146	.565*	-.524*	0.115	.898**	-0.118	.917**	-0.25	1

Figure S4. Heat map based on bivariate Spearman correlations in NALC (degrees of freedom differ, n=14 vs. 48). ** Correlation is significant at the 0.01 level (2- tailed). * Correlation is significant at the 0.05 level (2-tailed).
